# ADAR1 promotes cisplatin resistance in intrahepatic cholangiocarcinoma by regulating BRCA2 expression through A‐to‐I editing manner

**DOI:** 10.1111/cpr.13659

**Published:** 2024-05-21

**Authors:** Qi Liu, Chen‐Song Huang, Siyun Chen, Ying‐Qin Zhu, Xi‐Tai Huang, Guang‐Yin Zhao, Qiong‐Cong Xu, Yin‐Hao Shi, Wen Li, Ruizhi Wang, Xiao‐Yu Yin

**Affiliations:** ^1^ Department of Pancreato‐Biliary Surgery The First Affiliated Hospital of Sun Yat‐sen University Guangzhou China; ^2^ Key Laboratory of Stem Cells and Tissue Engineering (Sun Yat‐sen University) Ministry of Education Guangzhou China; ^3^ Department of Animal Experiment Center, The First Affiliated Hospital Sun Yat‐sen University Guangzhou China; ^4^ Laboratory of General Surgery, The First Affiliated Hospital Sun Yat‐sen University Guangzhou China; ^5^ Department of Laboratory Medicine, The First Affiliated Hospital Sun Yat‐sen University Guangzhou China; ^6^ Advanced Medical Technology Center, The First Affiliated Hospital, Zhongshan School of Medicine Sun Yat‐sen University Guangzhou China

## Abstract

Aberrant A‐to‐I RNA editing, mediated by *ADAR1* has been found to be associated with increased tumourigenesis and the development of chemotherapy resistance in various types of cancer. Intrahepatic cholangiocarcinoma (iCCA) is a highly aggressive malignancy with a poor prognosis, and overcoming chemotherapy resistance poses a significant clinical challenge. This study aimed to clarify the roles of *ADAR1* in tumour resistance to cisplatin in iCCA. We discovered that *ADAR1* expression is elevated in iCCA patients, particularly in those resistant to cisplatin, and associated with poor clinical outcomes. Downregulation of *ADAR1* can increase the sensitivity of iCCA cells to cisplatin treatment, whereas its overexpression has the inverse effect. By integrating RNA sequencing and Sanger sequencing, we identified *BRCA2*, a critical DNA damage repair gene, as a downstream target of *ADAR1* in iCCA. *ADAR1* mediates the A‐to‐I editing in *BRCA2* 3′UTR, inhibiting *miR‐3157‐5p* binding, consequently increasing *BRCA2* mRNA and protein levels. Furthermore, *ADAR1* enhances cellular DNA damage repair ability and facilitates cisplatin resistance in iCCA cells. Combining *ADAR1* targeting with cisplatin treatment markedly enhances the anticancer efficacy of cisplatin. In conclusion, *ADAR1* promotes tumour progression and cisplatin resistance of iCCA. *ADAR1* targeting could inform the development of innovative combination therapies for iCCA.

## Graphical abstract

*ADAR1* mediates the A‐to‐I editing in *BRCA2* 3′UTR, which further prevents *miR‐3157‐5p* binding to this region, resulting in upregulating *BRCA2* expression. Furthermore, *ADAR1* enhances cellular DNA damage repair ability and promotes cisplatin resistance in iCCA cells.

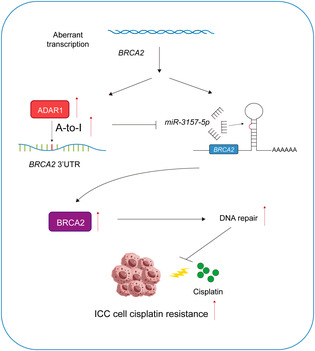

## 1 INTRODUCTION

Intrahepatic cholangiocarcinoma (iCCA) ranks as the second most frequent primary tumour in the liver and is known for its aggressive nature.^1^, ^2^, ^3^ The global incidence and mortality rates of iCCA have been rising due to its rapid progression and poor prognosis.^4^ Surgical resection remains the sole curative approach. However, most patients are diagnosed with unresectable disease, and there are limited treatment options for the high recurrence rate after surgery.^5^ Cisplatin‐based combination therapy is the standard first‐line treatment for patients with locally advanced or metastatic iCCA to improve outcomes.^2^ Yet, both intrinsic and acquired resistance to cisplatin significantly hinder treatment efficacy and lead to poor clinical results.

The characterized genomic and epigenomic changes in iCCA have indicated a significant level of tumour heterogeneity that may influence the therapeutic effects.^6^, ^7^ Genomic mutations on *BRCA2* have been reported to correlate with improved outcomes in cisplatin‐based therapy, resulting in better overall survival and clinical characteristics in iCCA patients.^8^, ^9^ Despite these findings, the impact of epigenetic modifications on *BRCA2* in iCCA and their influence on chemotherapy remains poorly understood.

RNA editing is one of the most common forms of epigenetic regulation at the post‐transcriptional level.^10^ Specifically, A‐to‐I RNA editing catalysed by the adenosine deaminase acting on RNA (*ADAR*) family occurs widely in mammals.^11^ *ADAR*s deaminatize adenosine to inosine (A‐to‐I) within double‐stranded RNAs (dsRNAs), which may further lead to alterations in pre‐mRNA processing, amino acid mutations, and instability of RNA secondary structure, thereby affecting multiple cellular processes and functions.^12^ In mammals, *ADAR1* is present in most human tissues and plays a major role in A‐to‐I RNA editing. Aberrant A‐to‐I RNA editing by *ADAR1* has been linked to increased tumourigenesis and chemotherapy resistance in various cancers, including breast cancer,^13^ hepatocellular carcinoma,^14^ and colorectal cancer.^15^ The specific role of *ADAR1* and its RNA‐edited targets in chemotherapy resistance in iCCA, however, remains elusive. Herein, we found *ADAR1* is upregulated in cisplatin chemoresistant iCCA tumours and is responsible for resistance to cisplatin in iCCA cells in an A‐to‐I editing manner. Mechanistically, *BRCA2* is identified as a direct target of *ADAR1* in iCCA cells. *ADAR1* editing of an adenine residue in the *BRCA2* 3′UTR blocks *miR‐3157‐5p* binding, thereby increasing *BRCA2* expression and enhancing DNA repair capabilities, which in turn promotes cisplatin resistance. Targeting *ADAR1*, combined with cisplatin treatment, has shown to amplify the anti‐tumour effects in a patient‐derived xenograft (PDX) model of iCCA with early postoperative recurrence.

## 2 MATERIALS AND METHODS

### 2.1 Patients' specimens

The study encompassed 152 iCCA patients, all histologically confirmed at the First Affiliated Hospital of Sun Yat‐sen University in Guangzhou, China. Of these, 128 patients, who had undergone radical hepatectomy and for whom complete clinical‐pathological characteristics and follow‐up data were available, were selected for correlation and prognosis analysis. The remaining 24 patients had received cisplatin as part of their chemotherapy regimen. All specimens were sourced from the Pathology Department of the First Affiliated Hospital of Sun Yat‐sen University. The efficacy of chemotherapy was assessed following the RECIST guidelines (version 1.1) for evaluating solid tumours, with evaluations conducted via computed tomography after two chemotherapy cycles. Patients demonstrating a complete response (CR) or partial response (PR) were classified as chemotherapy‐sensitive, whereas those with stable disease (SD) or progressive disease (PD) were categorized as chemotherapy‐resistant.^16^ The ethical approval for this study was granted by the First Affiliated Hospital of Sun Yat‐sen University, under the ethical code number [2021]712.

### 2.2 Analysis of public datasets

We investigated the mRNA expression levels of *ADAR1* and *BRCA2* and their correlation using the GEPIA2 database. Gene expression profiles GSE107943 and GSE119336 downloaded from the Gene Expression Omnibus (GEO) database were also analysed.

### 2.3 Cell culture and transfection

The human iCCA cell lines HuCC‐T1 and RBE were obtained from Cellcook Co., Ltd. (Guangzhou, China). Cells were cultured in RPMI 1640 medium (Gibco, USA) supplemented with 10% fetal bovine serum (Gibco, USA) at 37°C in a 5% CO_2_ incubator. The transfection of siRNA and shRNA were performed as described previously.^17^

### 2.4 Reagents and antibodies

The small interfering RNAs (siRNAs) targeting *BRCA2* and the short hairpin RNA (shRNA) sequences for *ADAR1* were acquired from HanYiBio Co. (Guangzhou, China) and Sangon Biotech Co., Ltd. (Shanghai, China), respectively. The siRNAs were designed by MISSION®. The *ADAR1*‐specific siRNAs for in vivo experiments were sourced from GenePharma Co., Ltd. (Suzhou, China). *ADAR1* overexpression and E/A plasmids were obtained from MiaoLingBio Co. (Wuhan, China). All miRNA mimics, and inhibitors were provided by RiboBio Co., Ltd. (Guangzhou, China). The *ADAR1* antibodies for immunohistochemistry (HPA003890) and Western blot analysis (mAb #81284) were purchased from Sigma and CST, respectively. Additionally, *BRCA2* (BA0668), Ki67 (ab156956), phosphorylated γ‐1H2AX (613415), and GAPDH (60004‐1‐IG) antibodies were sourced from Boster, Abcam, Biolegend, and Proteintech, respectively. The sequences of *ADAR1* shRNAs and *BRCA2* siRNAs are listed in Supplementary Table 1.

### 2.5 Western blot

Western blot analysis was conducted as previously described.^16^ iCCA cells were lysed using SDS loading buffer. Proteins were separated by SDS‐PAGE and transferred onto PVDF membranes (Merck Millipore, Germany). Membranes were blocked with 5% milk in TBST and incubated overnight at 4°C with primary antibodies targeting *ADAR1* or *BRCA2*. Following this, membranes were probed with appropriate secondary antibodies. Detection was achieved using ECL Western blot substrate (Proteintech, USA).

### 2.6 Dual‐luciferase reporter assay

HuCC‐T1 and RBE cells, seeded in 24‐well plates, were co‐transfected with miRNA mimic/inhibitors and reporter plasmids containing either wild‐type or mutant 3′‐UTR of *BRCA2* using Lipofectamine 2000. Forty‐eight hours post‐transfection, luciferase intensity was measured using the Dual‐Luciferase Reporter Assay System (Promega, USA). Firefly luciferase activity was normalized to Renilla luciferase activity.

### 2.7 In vivo assays

Female BALB/c nude mice, aged 4–5 weeks, were sourced from the Animal Experiment Center of the First Affiliated Hospital of Sun Yat‐Sen University for xenograft mouse models. A total of 1 × 10^7^ HuCC‐T1 or RBE cells, resuspended in 100 μL of phosphate‐buffered saline (PBS) mixed with Matrigel (1:1 ratio), from both sh*ADAR1* and control groups, were implanted into the right flank of the mice. Tumour volumes were measured every 3 days using a caliper. After 28 days, the mice were euthanized, and the tumours were photographed and weighed. Tumour volumes were calculated using the formula: *V* = (length × Width^2^)/2.

The patient‐derived xenograft (PDX) model was established using tissues from a patient with intrahepatic cholangiocarcinoma (iCCA) who relapsed within 6 months post R0 resection and cisplatin chemotherapy. The iCCA tissues were cut into 3 mm^3^ pieces and transplanted subcutaneously into the right flank of 5‐week‐old female B‐NDGR® mice, acquired from Biocytogen, Beijing, China. Once the xenografted tumours (Passage 1, P1) reached 1 cm^3^, they were fragmented and implanted into new B‐NDG® mice for serial transplantation (Passage 2–3, P2–3). This process allows for the propagation and expansion of the iCCA tumour cells in the PDX model. At P3, when tumour volumes reached 50 mm^3^, the BALB/c nu/nu mice were divided into four groups: siControl, si*ADAR1*, siControl + cisplatin, and si*ADAR1* + cisplatin. The siControl and si*ADAR1* groups received intratumoural injections of 3 nmol/L twice a week, while cisplatin was administered intraperitoneally at a dose of 4 mg/kg once a week. Tumour volumes were measured every 3 days. Twenty‐eight days post‐implantation, blood was collected from the inner canthus to assess levels of aminotransferase (AST), creatinine (CREA), alanine aminotransferase (ALT), and urea nitrogen (BUN). Subsequently, all mice were euthanized, and the tumours were photographed and weighed. All animal procedures were approved by the First Affiliated Hospital of Sun Yat‐Sen University ([2023] No. 204).

### 2.8 Immunohistochemistry (IHC)

The immunohistochemistry (IHC) staining assay was conducted as previously described.^16^ Two experienced pathologists independently evaluated the IHC scores, which were determined by both the staining intensity of protein expression (Negative, 0; Weak, 1; Moderate, 2; Strong, 3) and the percentage of the positive area (0%: 0; 1%–30%: 1; 31%–60%: 2; over 60%: 3). The final IHC score was calculated by multiplying these two values. Based on the median IHC score, the samples were categorized into high and low protein expression groups.

### 2.9 Real‐time quantitative polymerase chain reaction (RT‐qPCR)

Total RNA was extracted using Trizol reagent (Vazyme, China) and subsequently reverse transcribed to cDNA using HiScript II Q RT SuperMix for qPCR (R223‐01, Vazyme, China). Quantitative PCR (qPCR) was conducted using SYBR qPCR Master Mix (Q711‐02, Vazyme, China), following the manufacturer's instructions. Gene amplification and detection were performed on the QuantStudio 6 Flex Real‐Time PCR System. All RT‐qPCR primers were obtained from Sangon Biotech Co., Ltd. (Shanghai, China). Gene‐specific primers are listed in Supplementary Table 2.

### 2.10 RNA‐seq

Total RNA was extracted from HuCC‐T1 cells using Trizol reagent. The cDNA library was then constructed, and paired‐end sequencing was conducted by Novogene (Beijing, China). RNA integrity was assessed using the Agilent Bioanalyzer 2100. The resulting paired‐end reads were processed on the Illumina NovaSeq 6000 system and aligned to the human genome (hg38) using HISAT2. Differential expression genes (DEGs) were identified using the R package edgeR, with a significance threshold set at *p* < 0.05. Gene Set Enrichment Analysis (GSEA) was performed using the platform XianTao (https://www.xiantaozi.com/contact).

### 2.11 RNA editing analysis

RNA‐seq data were analysed using the REDItools package to quantify the nucleotide bases A, C, G, and T.^18^ The analysis focused on calculating the ratio of G to A at genomic sites where the DNA sequence is an A, but the transcriptome reflects a G. Only results with a ratio of G to A greater than or equal to 2 were considered. The reliability of this method was further verified by cloning individual sequences and performing Sanger sequencing. Direct sequencing of PCR products was carried out, and the editing frequency was calculated using SnapGene software.

### 2.12 Statistical analysis

Statistical analyses were conducted using SPSS version 22.0 and GraphPad Prism 9.0. Results were presented as mean ± standard deviation (SD) and compared using the *t*‐test. Disease‐free survival (DFS) and overall survival (OS) were calculated using the Kaplan–Meier method. Independent predictive factors were determined using the Cox proportional hazards model. Statistical significance was set at *p* < 0.05. Differences were considered statistically significant when *p* < 0.05 (two‐tailed) (*p < 0.05; **p < 0.01; ***p < 0.001).

## 3 RESULTS

### 3.1 *ADAR1* is up‐regulated in iCCA tissues, especially in chemoresistant iCCA, and is associated with poor clinical outcomes

**FIGURE 1:**
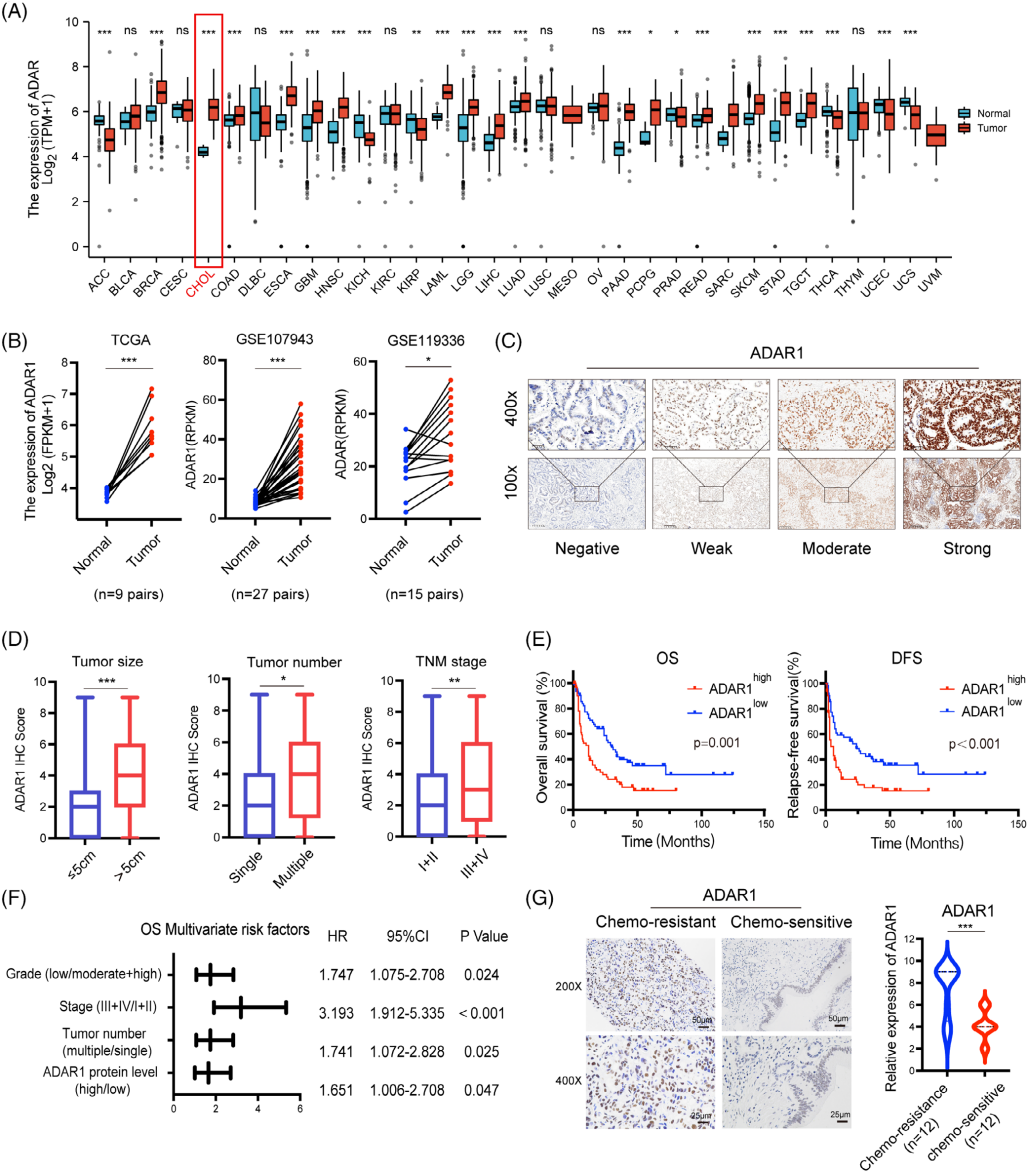
*ADAR1* is upregulated in iCCA tissues, particularly in chemoresistant iCCA, and is associated with poor clinical outcomes. (A) Comparison of *ADAR1* mRNA levels in tumour versus normal tissues across various cancers in TCGA database. (B) *ADAR1* mRNA expression in iCCA tumour and paracancerous tissues in TCGA and GEO databases. (C) Representative immunohistochemistry (IHC) images of *ADAR1* protein expression in iCCA tumours, highlighting variations in staining intensity. (D) Analysis of the relationship between *ADAR1* expression and tumour size, tumour number, and TNM stage in our in‐house cohort 128 iCCA cases. (E) Kaplan–Meier plots depicting overall survival (OS) and disease‐free survival (DFS) based on *ADAR1* IHC scoring. (F) Multivariate COX analysis of OS in our in‐house cohort 128 iCCA cases. (G) Representative IHC staining images and staining scores of *ADAR1* expression in the cisplatin chemo‐resistant group (*n* = 12) and chemo‐sensitive group (*n* = 12).

The expression of *ADAR1* mRNA was initially investigated across various cancer types using a pan‐cancer dataset (Figure 1A). Notably, *ADAR1* exhibited significant upregulation in cholangiocarcinoma tissues compared to adjacent non‐tumoural tissues. Further analysis of multiple public datasets, including TCGA and two GEO datasets, confirmed higher *ADAR1* expression in iCCA tumour tissues than in adjacent non‐cancerous tissues (Figure 1B). Additionally, we assessed the *ADAR1* protein expression of paraffin‐embedded specimens from 128 iCCA patients in our in‐house cohort by immunohistochemistry (IHC) assay, which revealed differential levels of *ADAR1* expression (Figure 1C). High *ADAR1* expression was positively correlated with larger tumour size, increased tumour number, and advanced tumour node metastasis (TNM) stage, but it showed no correlation with sex, age, or lymph node invasion (Figure 1D and Supplementary Table 3). Furthermore, Kaplan–Meier analysis demonstrated that higher *ADAR1* expression was associated with worse overall survival (OS) and disease‐free survival (DFS) in iCCA patients (Figure 1E). Multivariate regression analysis indicated *ADAR1* expression (HR = 1.651, 95%, *p* = 0.047)，tumour grade (HR = 1.747, *p* = 0.024), tumour stage (HR = 3.193, *p* < 0.001), and tumour number (HR = 1.741, *p* = 0.025) as independent predictors of overall survival in iCCA patients (Figure 1F and Table 1). Notably, *ADAR1* expression was significantly higher in chemotherapy‐resistant tumours than in chemotherapy‐sensitive tumours within our cohort (Figure 1G). These findings suggest that *ADAR1* may play an oncogenic role and promote cisplatin resistance in iCCA.

**TABLE 1:** Prognostic factor for DFS and OS of patients with intrahepatic cholangiocarcinoma.

	Overall survival			Disease‐free survival		
	HR	95% CI	*p*‐value	HR	95% CI	*p*‐value
Univariate						
Age (≥60/60)	0.925	0.605–1.415	0.719	0.915	0.598–1.400	0.682
Gender (male/female)	1.238	0.811–1.890	0.324	1.122	0.734–1.714	0.595
Number of tumour (multiple/single)	1.994	1.286–3.091	0.002	2.444	1.579–3.781	<0.001
Lymph node metastasis (+/−)	1.970	1.140–3.404	0.015	1.917	1.107–2.232	0.083
Distant metastasis (+/−)	1.870	1.139–3.071	0.013	3.016	1.857–4.898	<0.001
Stage (III + IV/I + II)	2.802	1.797–4.370	<0.001	3.667	2.316–5.805	<0.001
Grade (low/moderate + high)	1.681	1.067–2.650	0.025	1.684	1.068–2.655	0.025
*ADAR1* protein level (high/low)	2.037	1.325–3.130	0.001	2.131	1.386–3.278	<0.001
Multivariate						
Number of tumour (multiple/single)	1.741	1.072–2.828	0.025	2.041	1.259–3.310	0.004
Lymph node metastasis (+/−)	1.550	0.741–3.241	0.244	0.388	0.167–0.905	0.029
Distant metastasis	0.552	0.267–1.143	0.109	2.072	0.974–4.408	0.058
Stage (III + IV/I + II)	3.193	1.912–5.335	<0.001	3.990	2.339–6.807	<0.001
Grade (low/moderate + high)	1.747	1.075–2.838	0.024	1.994	1.220–3.261	0.006
*ADAR1* protein level (high/low)	1.651	1.006–2.708	0.047	1.611	0.993–2.616	0.054

### 3.2 *ADAR1* enhances iCCA resistance to cisplatin via A‐to‐I RNA editing

**FIGURE 2:**
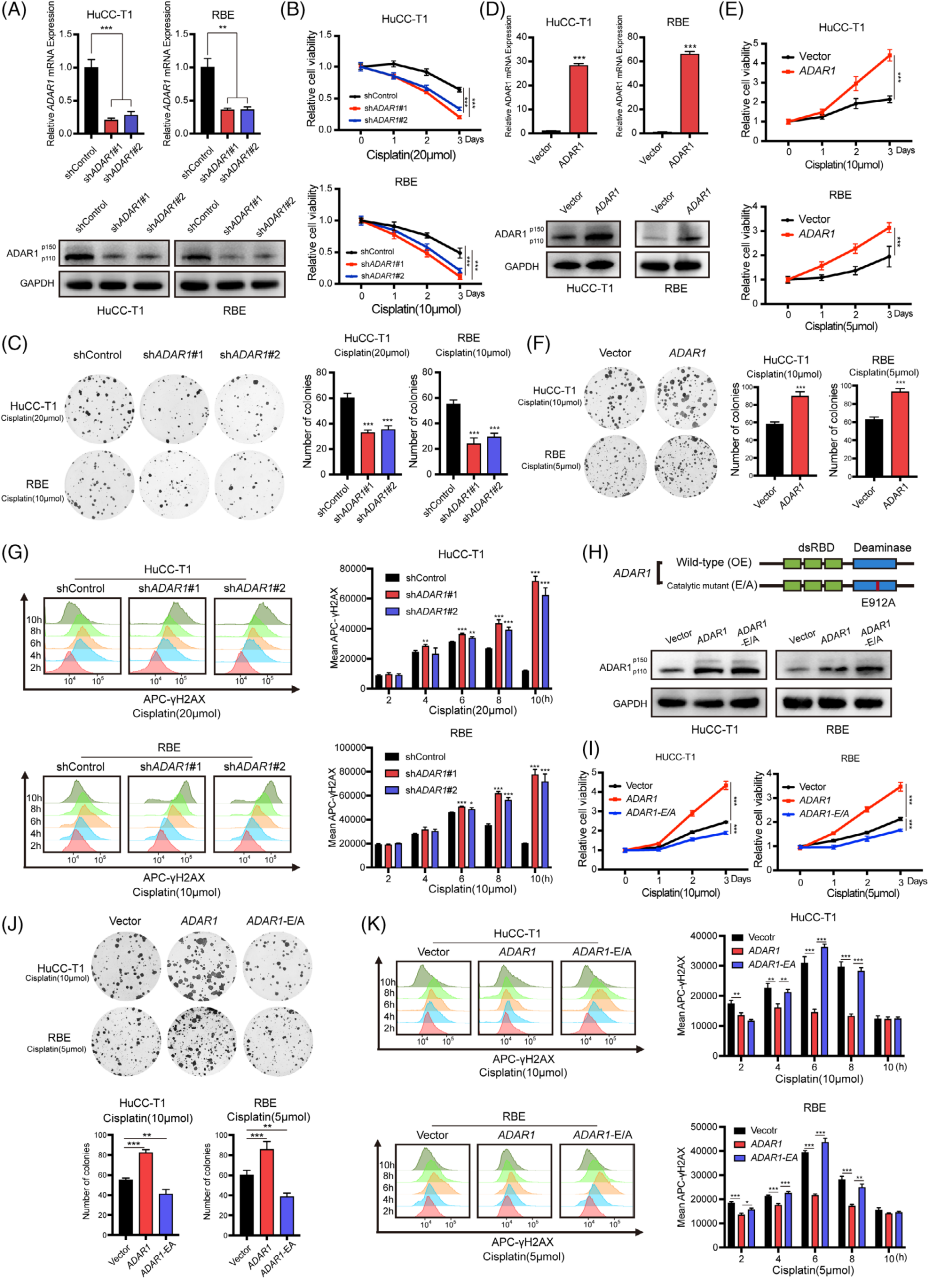
*ADAR1* promotes iCCA resistance to cisplatin via A‐to‐I RNA editing. (A) *ADAR1* mRNA and protein levels in sh*ADAR1* and control iCCA cells, assessed by RT‐qPCR and Western blot. (B) Cell viability of sh*ADAR1* and control groups post‐cisplatin treatment (20 or 10 μM, respectively). (C) Colony formation in sh*ADAR1* and control groups post‐cisplatin exposure (20 or 10 μM, respectively). (D) mRNA and protein expression of *ADAR1* in wild‐type *ADAR1*‐overexpressing (OE) and control iCCA cells. (E) Cell viability in wild‐type *ADAR1*‐OE and control groups post‐cisplatin treatment (10 or 5 μM, respectively). (F) Colony formation assay results of the wild‐type *ADAR1*‐OE and control groups in iCCA cells after cisplatin treatment (10 or 5 μM, respectively). (G) Flow cytometry and γ‐H2AX fluorescence intensity in sh*ADAR1* and control groups post‐cisplatin treatment (20 or 10 μM, respectively). (H) Schematic and transfection efficiency validation of wild‐type and catalytic mutant (E/A) ADAR1 plasmids via Western blot. (I) Cell viability in *ADAR1*‐OE, E/A, and control groups post‐cisplatin treatment (10 or 5 μM, respectively). (J) Colony formation assay results of the *ADAR1*‐OE group, E/A group and control group in iCCA cells after cisplatin treatment (10 or 5 μM, respectively). (K) Representative flow cytometry analysis and γ‐H2AX mean fluorescent intensity of the *ADAR1*‐OE group, E/A group and control group in iCCA cells after cisplatin treatment (10 or 5 μM, respectively).

To better understand the biological function of *ADAR1* in iCCA upon cisplatin treatment, we established stable *ADAR1* knockdown cell lines using shRNA lentivirus infection and assessed cell response after cisplatin treatment. The efficiency of *ADAR1* knockdown at both mRNA and protein levels was confirmed using qPCR and western blot (Figure 2A), respectively. As expected, knocking down *ADAR1* increased the sensitivity to cisplatin treatment in HuCC‐T1 and RBE cells, resulting in more pronounced inhibition of cell proliferation and colony formation compared to the control group (Figure 2B,C). Mammalian cells express two *ADAR1* isoforms: the interferon‐induced cytoplasmic p150, and the constitutively expressed, nuclear‐localized p110.^19^, ^20^ In iCCA cells, the expression of p150 is low. We therefore generated HuCC‐T1 and RBE cells exogenously expressing p110 *ADAR1* (Figure 2D). Conversely, ectopic expression of *ADAR1* conferred resistance to cisplatin in these cells (Figure 2E,F). Further, we quantified cisplatin‐induced DNA damage using flow cytometry, finding that the downregulation of *ADAR1* increased DNA damage in iCCA cells (Figure 2G). During a 10‐h drug treatment, while DNA repair occurred in control cells, it was impaired in *ADAR1*‐deficient cells.

*ADAR1* catalyses the deamination of adenosine to inosine (A‐to‐I) in double‐stranded RNA. To explore the effects of disrupting this activity, we introduced the E912A mutation into iCCA cell lines, resulting in a dysfunctional *ADAR1* protein (Figure 2H). We then performed cell proliferation and colony formation assays under cisplatin treatment to evaluate the mutation's impact. Overexpression of wild‐type *ADAR1* in iCCA cells led to increased cell proliferation and colony numbers post‐treatment (Figure 2I,J). Conversely, cells expressing the E912A mutant *ADAR1* exhibited reduced tumour growth and colony formation relative to controls. Analysis of DNA damage revealed that γ‐H2AX mean fluorescent intensity remained stable in cells overexpressing *ADAR1* but changed dramatically in the E912A mutant and control groups (Figure 2K). These results demonstrate that *ADAR1*'s role in modulating iCCA resistance to cisplatin is dependent on its A‐to‐I editing function.

### 3.3 *ADAR1* regulates *BRCA2* expression through A‐to‐I RNA editing

**FIGURE 3:**
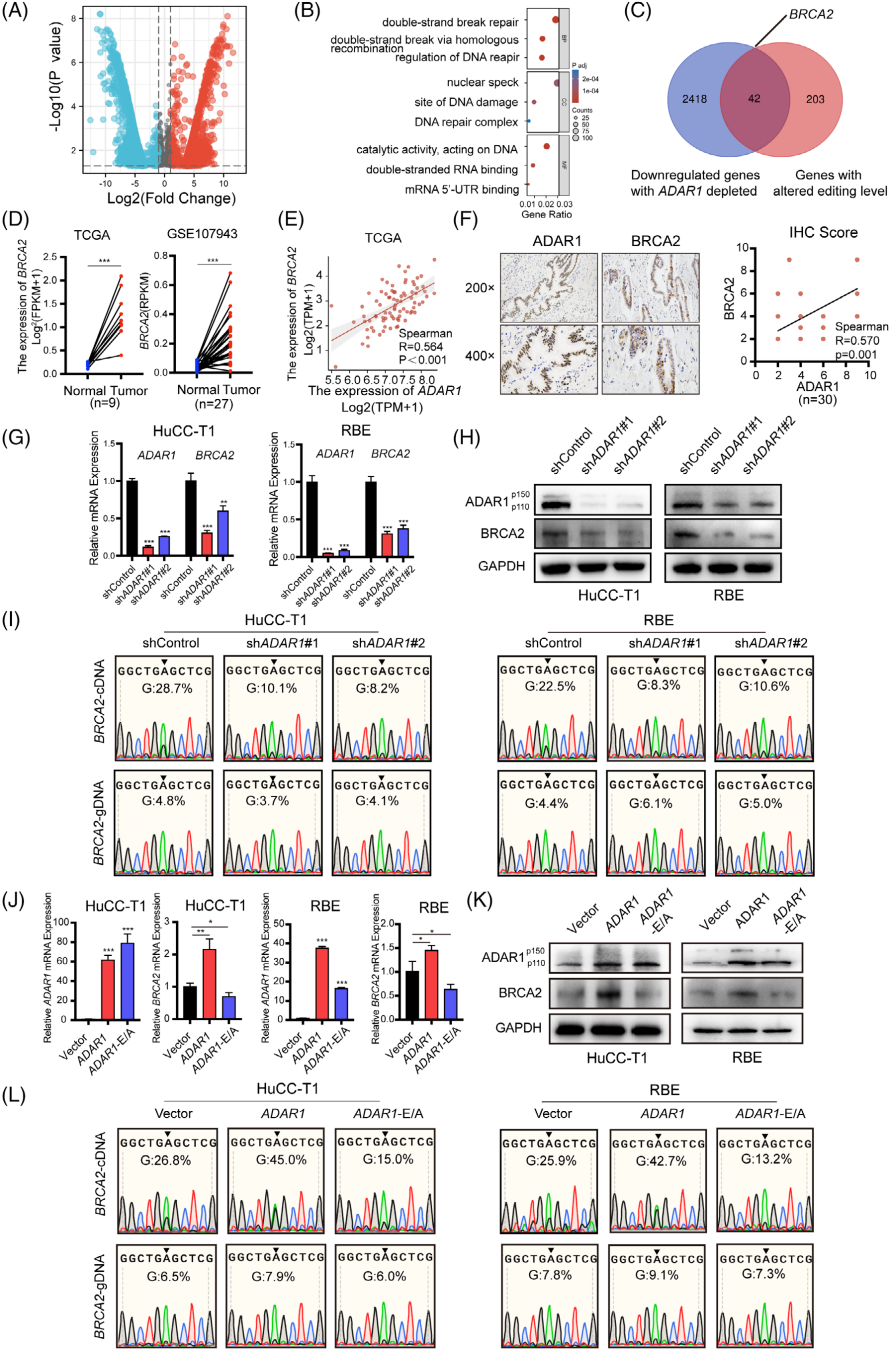
*ADAR1* regulates *BRCA2* expression through A‐to‐I RNA editing. (A) Volcano plot illustrating differential gene expression obtained by RNA sequencing of HuCC‐T1 with stable knockdown of *ADAR1*, down‐regulated genes are highlighted in blue, and up‐regulated genes in red. (B) GO enrichment analysis for differentially expressed genes (DEGs) in sh*ADAR1* and shControl cells, covering biological processes (BP), cellular components (CC), and molecular functions (MF). (C) Venn diagram showing the overlap between genes downregulated after *ADAR1* knockdown and genes exhibiting A‐to‐I RNA editing in the 3′UTR. (D) Comparison of *BRCA2* mRNA expression between iCCA tumour and paracancerous tissues in TCGA and GSE107943 datasets. (E) Correlation analysis between *ADAR1* and *BRCA2* mRNA expression in TCGA iCCA tumour and paracancerous tissues. (F) Representative IHC staining images and protein correlation analysis between *ADAR1* and *BRCA2* expression in the in‐house cohort (*n* = 30). (G) Assessment of the *ADAR1* and *BRCA2* mRNA expression of the sh*ADAR1* group and shControl group in iCCA cells via RT‐qPCR. (H) Evaluation of *ADAR1* and *BRCA2* protein levels of iCCA cells from shADAR1 and shControl groups using western blot. (I) Sanger sequencing chromatograms of *BRCA2* A‐to‐I RNA editing sites in iCCA cells from sh*ADAR1* and shControl groups. (J) RT‐qPCR analysis of *ADAR1* and *BRCA2* mRNA expression in iCCA cells from *ADAR1*‐OE group, E/A group, and control group. (K) Western blot analysis of the *ADAR1* and *BRCA2* protein levels in iCCA cells from *ADAR1* OE group, E/A group, and control group. (L) Sanger sequencing chromatograms of *BRCA2* A‐to‐I RNA editing sites in iCCA cells from *ADAR1‐*OE group, E/A group and control group.

To elucidate *ADAR1*'s role in iCCA chemoresistance to cisplatin, we performed RNA sequencing on *ADAR1*‐deficient cells and control cells. A total of 4373 differentially expressed genes (DEGs) were identified in *ADAR1* knockdown HuCC‐T1 cells compared to the control group (Figure 3A), with a significant downregulation of 2460 genes. Subsequent gene ontology (GO) analysis revealed enrichment in terms related to DNA repair and double‐strand break repair (Figure 3B). Considering *ADAR1*'s role in iCCA progression through A‐to‐I RNA editing, our analysis focused on downregulated genes with altered A‐to‐I editing levels post‐*ADAR1* knockdown. We discovered 245 genes with modified A‐to‐I editing rates in the 3′ untranslated regions (UTRs) by comparing their genomic DNA. Integrating RNA‐seq and Sanger sequencing data revealed 42 intersecting genes (Figure 3C and Supplementary Table 4). We further identified tumour‐related genes through literature review and assessed their RNA expression in *ADAR1*‐deficient cells using RT‐qPCR. Notably, *BRCA2* showed the most significant RNA expression reduction, aside from *ADAR1* (Supplementary Figure 1). Literature review underscored *BRCA2*'s role in DNA damage repair and its critical function in cancer chemoresistance.^21^, ^22^ Analysis of *BRCA2* expression in TCGA and GEO datasets revealed its upregulation in iCCA tumours compared to non‐tumoural tissues (Figure 3D). A positive correlation between *ADAR1* and *BRCA2* expression was observed in both iCCA tumours and non‐tumoural tissues from TCGA (Figure 3E), which was corroborated in our iCCA cohort via IHC staining (*n* = 30, Figure 3F). Additionally, we observed a significant reduction in *BRCA2* mRNA and protein levels following *ADAR1* knockdown (Figure 3G,H). Collectively, these findings suggest that *BRCA2* is a downstream target of *ADAR1*, implicating *ADAR1* in promoting iCCA resistance to cisplatin by modulating *BRCA2* expression.

We further investigated the impact of *ADAR1* knockdown on A‐to‐I RNA editing rates in the *BRCA2* gene across iCCA cells. The base editing rate at the cDNA level was reduced in *ADAR1* knockdown cells compared to the control group, whereas it remained consistent at the gDNA level between both groups (Figure 3I). Additionally, overexpression of the *ADAR1* E912A mutant resulted in decreased *BRCA2* expression at both mRNA and protein levels in HuCC‐T1 and RBE cells, relative to cells overexpressing the wild‐type *ADAR1* (Figure 3J,K). This reduction in expression correlates with a significant decrease in the base editing rate of *BRCA2* mRNA in cells expressing the *ADAR1* E912A mutant compared to those with wild‐type *ADAR1* (from 45% to 15% in HuCC‐T1 and from 42.7% to 13.2% in RBE) (Figure 3L). These findings suggest that *ADAR1* modulates *BRCA2* in an A‐to‐I RNA editing‐dependent manner.

### 3.4 *ADAR1* promotes cisplatin resistance of iCCA cells through *BRCA2*

**FIGURE 4:**
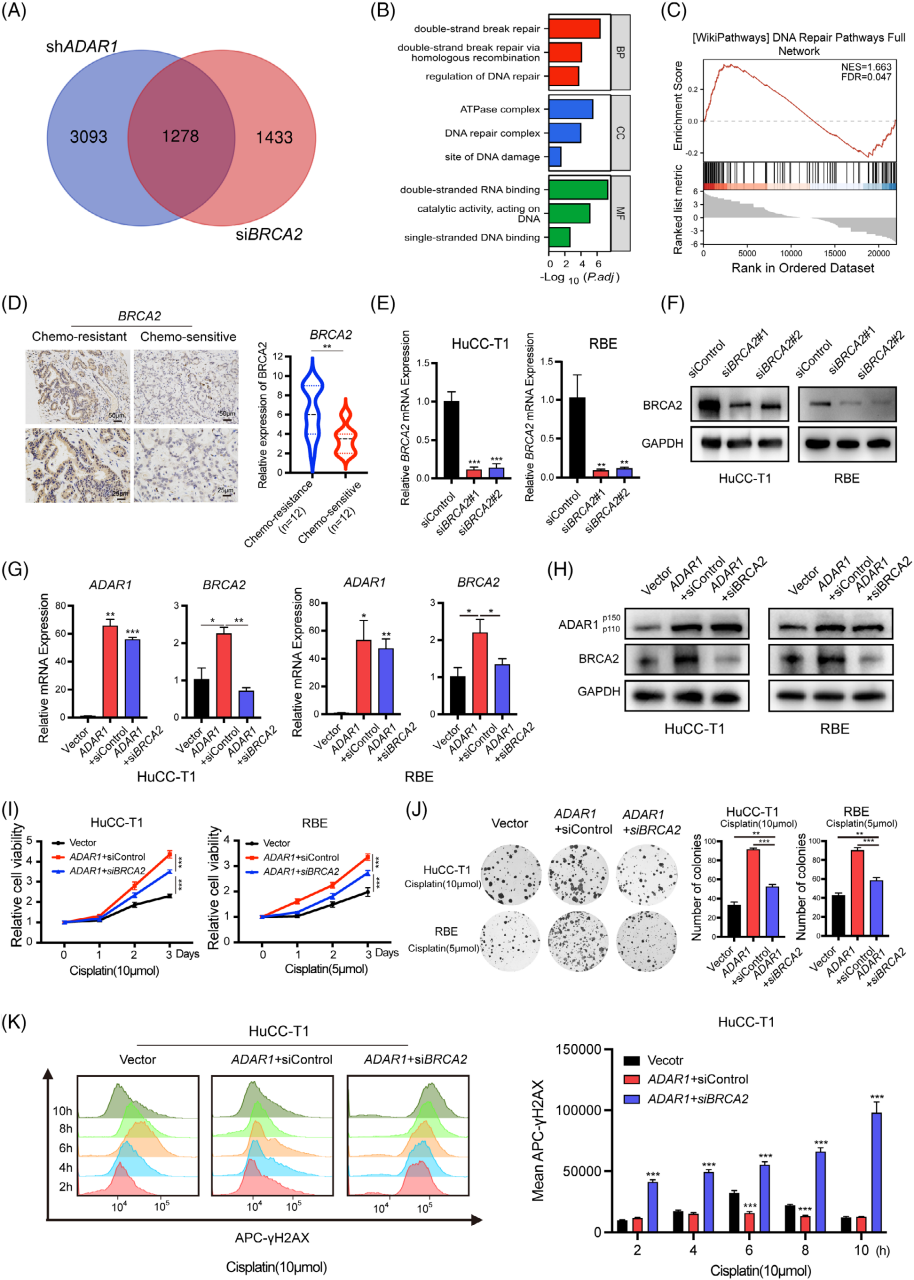
*ADAR1* promotes cisplatin resistance in iCCA cells via *BRCA2*. (A) Venn diagram illustrating the overlap in differentially expressed genes (DEGs) between sh*ADAR1* and si*BRCA2* RNA‐seq. (B) Gene Ontology (GO) enrichment analysis of overlapping DEGs from sh*ADAR1* and si*BRCA2* RNA‐seq, including biological processes (BP), cellular components (CC), and molecular functions (MF). (C) Gene Set Enrichment Analysis (GSEA) of overlapping DEGs between sh*ADAR1* and si*BRCA2* RNA‐seq. (D) Representative immunohistochemistry (IHC) staining images and scoring of *BRCA2* in cisplatin‐resistant (*n* = 12) and sensitive groups (*n* = 12). (E) Validation of *BRCA2* mRNA expression post‐*BRCA2 silencing* in iCCA cells using RT‐qPCR. (F) Validation of *BRCA2* protein expression post‐*BRCA2* silencing in iCCA cells using Western blot. (G) Measurement of *BRCA2* mRNA levels in vector, *ADAR1* + siControl, and *ADAR1* + si*BRCA2* groups in iCCA cells by RT‐qPCR. (H) Measurement of *BRCA2* protein levels in vector, *ADAR1* + siControl, and *ADAR1* + si*BRCA2* groups in iCCA cells by Western blot. (I) Cell viability curves for vector, *ADAR1* + siControl, and *ADAR1* + si*BRCA2* groups in iCCA cells post‐cisplatin treatment (10 or 5 μM). (J) Colony formation assay outcomes for vector, *ADAR1* + siControl, and *ADAR1* + si*BRCA2* groups in iCCA cells treated with cisplatin (10 or 5 μM). (K) Representative flow cytometry analysis and γ‐H2AX mean fluorescent intensity in vector, *ADAR1* + siControl, and *ADAR1* + si*BRCA2* groups in HuCC‐T1 cells post‐cisplatin treatment (10 μM).

*BRCA2* is crucial in the homologous recombination pathway for double‐strand DNA repair.^21^ Our RNA‐seq data (Figure 4A) revealed that genes regulated by both *ADAR1* and *BRCA2* are clustered in biological processes related to DNA repair (Figure 4B). Furthermore, genes associated with DNA repair pathways are significantly downregulated when *BRCA2* is deficient (Figure 4C). Recent research in ovarian cancer has indicated that the mutation status of the *BRCA* gene can predict the response to treatment.^23^ Clinical trials evaluating *BRCA* mutation status have demonstrated enhanced responses to platinum‐based chemotherapies, underscoring the pivotal role of *BRCA2* in cancer treatment.^24^, ^25^ Additionally, in our cohort of iCCA patients treated with cisplatin, we noted a significant increase in *BRCA2* expression in chemotherapy‐resistant tumours compared to those sensitive to chemotherapy (Figure 4D). These observations led us to hypothesize that the *ADAR1/BRCA2* axis might be contributing to cisplatin resistance in iCCA. To explore the role of the *ADAR1/BRCA2* axis in promoting chemoresistance, we used siRNAs to inhibit *BRCA2* in control cells and cells expressing wild‐type *ADAR1* (Figure 4E–H). The downregulation of *BRCA2* partially restored sensitivity to cisplatin in cells overexpressing wild‐type *ADAR1* (Figure 4I,J). the capacity for DNA damage repair was also diminished following *BRCA2* knockdown in cells overexpressing wild‐type *ADAR1* (Figure 4K).

### 3.5 A‐to‐I RNA editing on *BRCA2* inhibits *miR‐3157‐5p* binding to its 3′UTR and regulates the level of *BRCA2* expression

**FIGURE 5:**
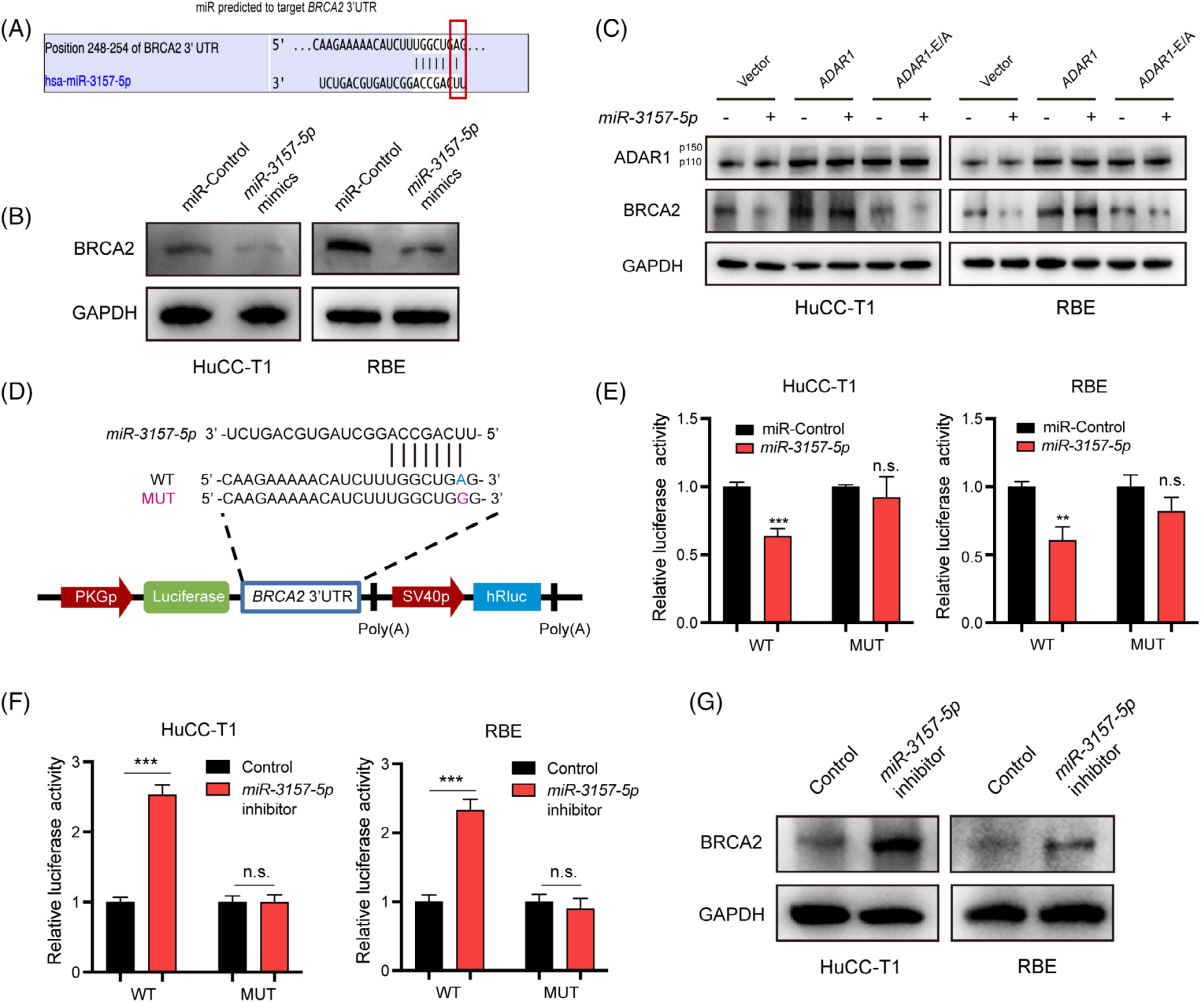
A‐to‐I RNA editing on *BRCA2* mediated by *ADAR1* inhibits *miR‐3157‐5p* binding to its 3′UTR and regulates the level of *BRCA2* expression. (A) TargetScan predicts miRNAs that may bind to the A‐to‐I edited site in the *BRCA2* 3′UTR. (B) Assessed *BRCA2* protein expression in iCCA cells transfected with the *miR‐3157‐5p* mimic or control using Western blot. (C) Evaluated *ADAR1* and *BRCA2* protein expressions in vector, *ADAR1*‐OE, and *ADAR1*‐EA groups in iCCA cells transfected with *miR‐3157‐5p* mimics using Western blot. (D) Schematic representation of the *BRCA2* 3′UTR luciferase reporter plasmid, including both wildtype (WT) and mutant (MUT) versions. (E) Dual luciferase reporter assay results for the *BRCA2*‐WT and *BRCA2*‐MUT groups in iCCA cells co‐transfected with *miR‐3157‐5p* mimics or miR‐Control. (F) Dual luciferase reporter assay results for the *BRCA2*‐WT and *BRCA2*‐MUT groups in iCCA cells co‐transfected with *miR‐3157‐5p* inhibitor or control. (G) Assessed *BRCA2* protein expression in iCCA cells transfected with the *miR‐3157‐5p* inhibitor or control.

The A‐to‐I RNA edited site within *BRCA2* is located in the 3′UTR (Figure 5A), a well‐known region that influences gene expression through mRNA‐based processes such as mRNA localization, stability, and translation.^26^ For instance, mRNA‐bound by miRNAs can either be degraded or preserved, rather than being immediately translated. Therefore, we conducted an analysis to scan the miRNA that binds to the base‐edited region of *BRCA2*, and *miR‐3157‐5p* was predict as a potential regulator of *BRCA2* expression(Figure 5A).

Subsequently, we transfected *miR‐3157‐5p* mimics to investigate their effects on regulating *BRCA2* expression and iCCA progression. We noted a reduction in the protein level of *BRCA2* after transfection with *miR‐3157‐5p* mimics (Figure 5B). Additionally, we evaluated the effect of *miR‐3157‐5p* in iCCA cells expressing either wild‐type or E912A mutant *ADAR1*. Western blot analysis revealed that transfection with miR‐3157‐5p did not alter *BRCA2* expression in cells with wild‐type *ADAR1*. However, in cells expressing the E912A mutant, *miR‐3157‐5p* transfection led to decreased *BRCA2* protein levels in both HuCC‐T1 and RBE cells (Figure 5C). This was most likely due to the majority of pre‐mRNAs of *BRCA2* being mutated by the overexpressed wild‐type *ADAR1*, preventing their binding with *miR‐3157‐5p*. Conversely, with the E912A‐mutant *ADAR1*, the majority of the *BRCA2*‐3′UTR remained unedited, allowing *miR‐3157‐5p* to bind to the *BRCA2* mRNA and promote its degradation.

To further elucidate this regulatory mechanism via miRNA binding, we utilized the Dual‐Luciferase reporter system with *BRCA2*'s 3′UTR, both with and without the A‐to‐G mutation (Figure 5D). Expression of *miR‐3157‐5p* in iCCA cells significantly reduced the luciferase signal in the *BRCA2*‐mutated group compared to the wild‐type group (Figure 5E). Conversely, inhibition of *miR‐3157‐5p* markedly increased luciferase activity (Figure 5F) and *BRCA2* expression (Figure 5G) in the wild‐type group, but had no effect in the mutated group. These results illustrate that *ADAR1*, by blocking *miR‐3157‐5p* binding to the base‐edited site of *BRCA2*, regulates the expression of *BRCA2*.

### 3.6 Enhancing anti‐tumour efficacy of cisplatin in iCCA PDX models via *ADAR1* targeting

**FIGURE 6:**
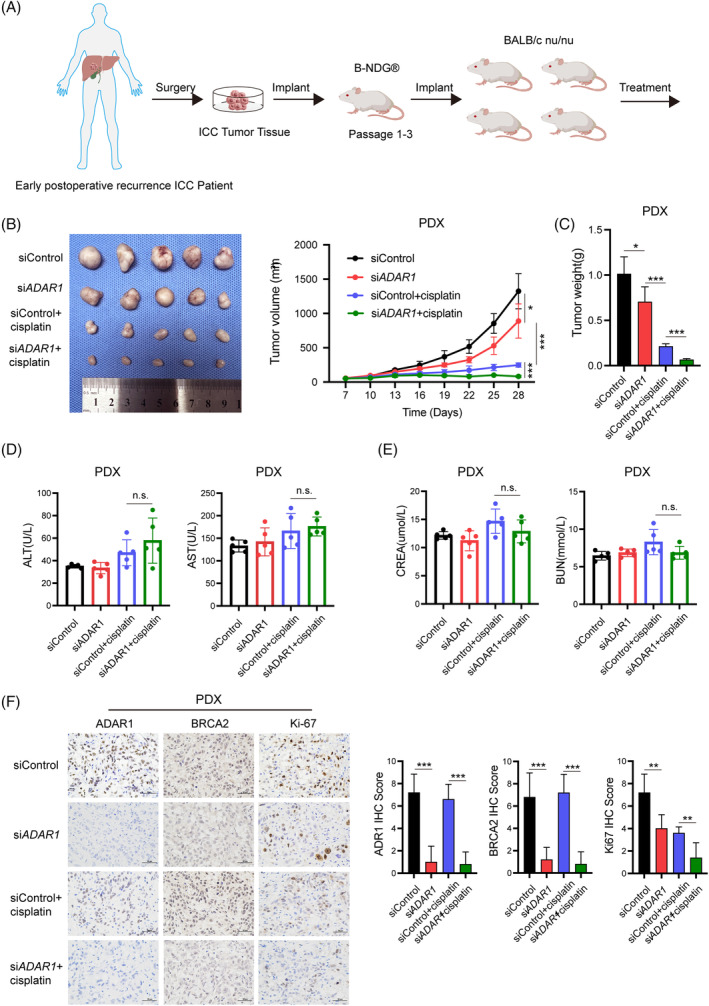
Enhancing anti‐tumour efficacy of cisplatin in iCCA PDX models via *ADAR1* targeting. (A) Graphic depiction of constructing a cisplatin‐resistant iCCA PDX model. (B) PDX tissues were randomly assigned into four groups and treated as follows: siControl (3 nmol per mouse, twice a week), si*ADAR1* (3 nmol per mouse, twice a week), siControl plus cisplatin (siControl, 3 nmol per mouse, twice a week, and cisplatin, 4 mg/kg, once a week), and si*ADAR1* plus cisplatin (si*ADAR1*, 3 nmol per mouse, twice a week, and cisplatin, 4 mg/kg, once a week) over 21 days. Tumour growth curves for each group are displayed. (C) Tumour weights for the siControl, si*ADAR1*, siControl + cisplatin, and si*ADAR1* + cisplatin groups were measured post‐euthanasia. (D) Serum ALT and AST levels, indicators of liver function, were measured in the siControl, si*ADAR1*, siControl + cisplatin, and si*ADAR1* + cisplatin groups. (E) Serum creatinine (CREA) and blood urea nitrogen (BUN), indicators of kidney function, were measured in the siControl, si*ADAR1*, siControl + cisplatin, and si*ADAR1* + cisplatin groups. (F) Representative immunohistochemistry (IHC) staining and IHC scores for *ADAR1*, *BRCA2*, and *Ki67* in tumours from the siControl, si*ADAR1*, siControl + cisplatin, and si*ADAR1* + cisplatin groups.

To explore the role of *ADAR1* in modulating the response to cisplatin in vivo, we employed an iCCA patient‐derived xenograft (PDX) model, which originated from a patient who experienced early postoperative recurrence after receiving cisplatin and gemcitabine chemotherapy (Figure 6A). This patient had undergone surgical resection but relapsed within 6 months. Tumours from passage 3 were implanted into 5‐week‐old female BALB/c nude mice. When the tumour volume reached approximately 50 mm^3^, the mice were randomly assigned to four different treatment groups. The group receiving combined treatment of *ADAR1* siRNA and cisplatin showed a significant reduction in tumour volume (Figure 6B) and tumour weight (Figure 6C) compared to the groups receiving either treatment alone. Importantly, the combined treatment did not result in significant liver (Figure 6D) or kidney toxicity (Figure 6E). IHC staining revealed a marked decrease in the expression levels of *ADAR1*, *BRCA2*, and *Ki67* in the tumours of the combination treatment group (Figure 6F), suggesting that targeting *ADAR1* alongside cisplatin treatment can significantly impair cell proliferation. Collectively, the findings from the PDX model demonstrated that targeting *ADAR1* promotes the anti‐tumour efficacy of cisplatin in iCCA.

## 4 DISCUSSION

**FIGURE 7:**
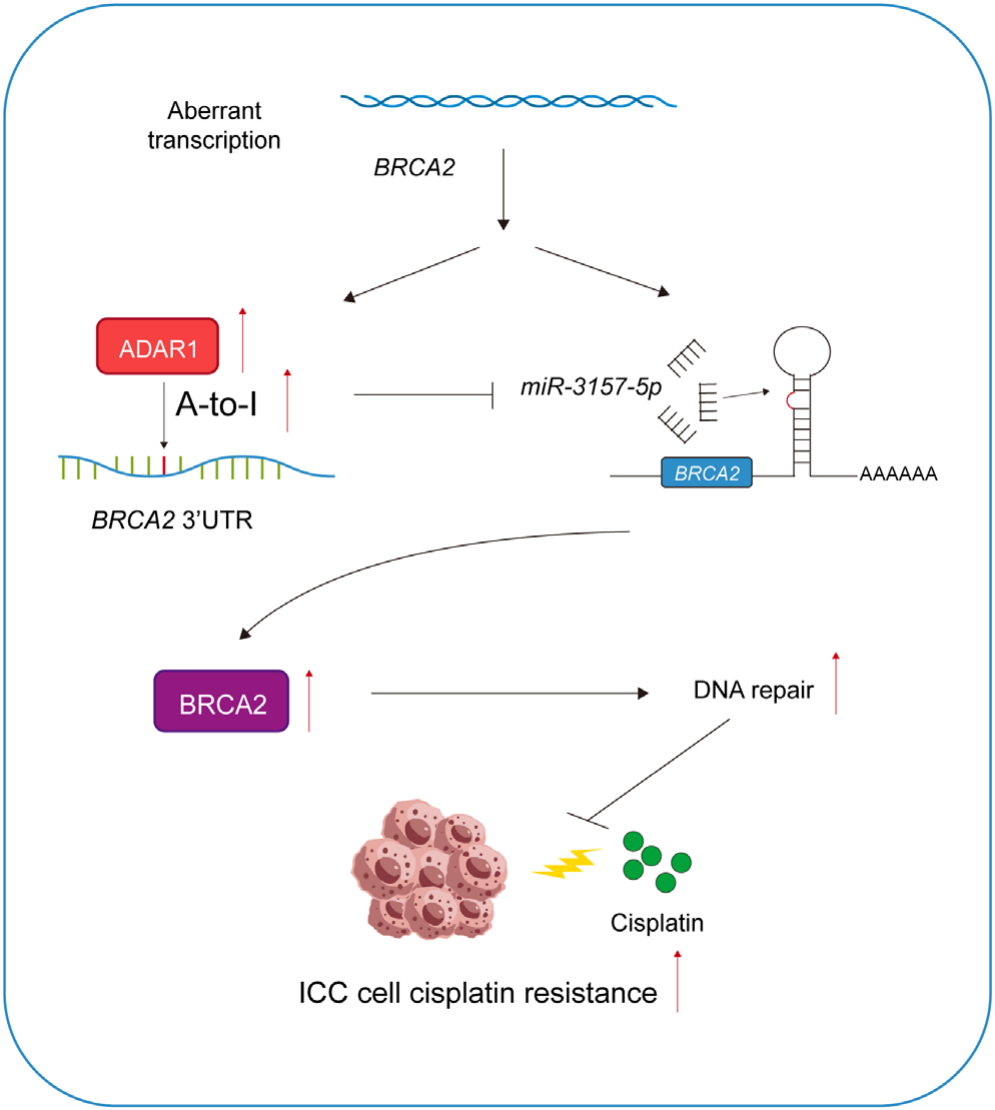
Schematic diagram illustrating the function and mechanism of *ADAR1* in iCC.

iCCA is a highly aggressive malignancy characterized by frequent primary progression and metastasis. Despite advancements in therapeutic strategies, the overall survival rate for iCCA patients remains poor.^27^ Chemotherapy serves as the primary treatment for patients ineligible for surgical resection, yet chemotherapy resistance in iCCA is a pressing issue demanding urgent attention. Numerous studies have underscored the importance of epigenetic regulation in drug resistance.^28^ For example, the m6A methyltransferase *METTL3* mediates the expression of a histone modifier *EZH2* in an m6A‐dependent manner under temozolomide treatment in glioblastoma. This interplay of m6A modification and H3K27ac modification ultimately leads to temozolomide‐resistance in glioblastoma.^29^ In iCCA, our previous research found that m6A modification regulates iCCA progression via the *METTL3‐YTHDF2* axis.^30^ Another study further uncovers the role of m6A modification and its reader protein *YTHDF2* in cisplatin resistance of iCCA.^16^ Here, we propose a novel epigenetic mechanism in iCCA cisplatin resistance, where *ADAR1* mediates cisplatin resistance in iCCA through the regulation of *BRCA2* via A‐to‐I RNA editing. Aberrant expression of *ADAR1* leads to a high frequency of A‐to‐I RNA editing in the *BRCA2* 3′UTR, which prevents mRNA degradation by inhibiting *miR‐3157‐5p* binding. Elevated *BRCA2* protein level enhances DNA damage repair in iCCA cells, leading to cisplatin resistance (Figure 7).

As the downstream target of *ADAR1*, *BRCA2* is a well‐known component involved in DNA repair, primarily regulating the recombinase enzyme *RAD51* to form DNA recombinase complexes. This process is essential for pairing homologous sequences in the repair of DNA double‐strand breaks.^31^ In iCCA, about 1%–3% of patients carry germline mutations in the *BRCA2* gene.^32^ Clinically, iCCA patients with genomic *BRCA2* mutations exhibit better responses to cisplatin treatment compared to those without such mutations.^9^ However, the majority of iCCA patients do not harbour genomic *BRCA2* mutations and do not benefit from cisplatin‐based chemotherapy. In fact, some patients develop resistance to cisplatin, which may be attributed to posttranscriptional regulation of *BRCA2*.

*ADAR1* acts as an upstream regulator of *BRCA2*, modulating its expression through A‐to‐I RNA editing. We observed upregulation of both *ADAR1* and *BRCA2* in chemo‐resistant tumours compared to chemo‐sensitive tumours. Additionally, the knockdown of *ADAR1* sensitizes iCCA cells to cisplatin. In our PDX model, targeting *ADAR1* with siRNA significantly attenuated tumour growth compared to the control group. Furthermore, si*ADAR1* sensitized iCCA tumours to cisplatin and enhanced the anti‐tumour effect of cisplatin in combined treatment. These findings suggest the possibility that iCCA patients may also benefit from a cisplatin‐based treatment strategy by targeting *ADAR1*. It is noteworthy that a selective *ADAR1* inhibitor is now available for cancer research.^33^ Hopefully, the development and clinical translation of a combined therapeutic strategy targeting *ADAR1* and cisplatin will be realized soon.

## 5 CONCLUSIONS

In conclusion, our study elucidates the impact of *ADAR1* on the response to cisplatin treatment in iCCA. Abnormal *ADAR1* expression promotes cisplatin resistance in iCCA by regulating *BRCA2* expression through A‐to‐I RNA editing. These findings underscore the significance of *ADAR1*‐mediated A‐to‐I RNA editing and offer novel insights into the optimization of cisplatin‐based treatments for iCCA.

## AUTHOR CONTRIBUTIONS

YXY conceived the study. YXY, WRZ and LW designed and guided the study. LQ, HCS, CSY and ZGY performed the in vitro and in vivo experiments. HCS, ZYQ, XQC and SYH conducted the statistical analysis. LQ and CSY wrote the first draft of the paper. YXY, WRZ and LW revised the manuscript. All authors read and approved the final version.

## FUNDING INFORMATION

This work was supported by grants from National Natural Science Foundation of China (Grant Nos. 82072644, 82002501, 81672417, 81972750, 31871479, 82203105), the Guangdong Basic and Applied Basic Research Foundation (Grant No. 2021A1515010123) and Guangzhou Science and Technology Project (Grant No. 202201010927).

## CONFLICT OF INTEREST STATEMENT

The authors declare no conflicts of interest.

## Supporting information


**Supplementary Figure 1.** RNA expression of overlapping genes between downregulated genes after knockdown *ADAR1* and genes with altered A‐to‐I editing level.
**Supplementary Table 1.** Sequences of shRNAs, and siRNAs used for experiments in this study.
**Supplementary Table 2.** Primers sequences of gene in this study.
**Supplementary Table 3.** Correlation between ADAR1 expression and iCCA in 128 iCCA patients.
**Supplementary Table 4.** List of downregulated genes after knockdown *ADAR1*, genes with altered A‐to‐I editing level and overlapping genes.

## Data Availability

The data that support the findings of this study are available from the corresponding author upon reasonable request.
