# Supplementary material for: ADAR1 promotes cisplatin resistance in intrahepatic cholangiocarcinoma by regulating BRCA2 expression through A‐to‐I editing manner
Source: Cell Prolif. 2024 May 21;57(10):e13659. doi: 10.1111/cpr.13659 (PMC11471395; doi:10.1111/cpr.13659)
Supplement: Supplementary file 1 — Supplementary Figure 1. RNA expression of overlapping genes between downregulated genes after knockdown ADAR1 and genes with altered A‐to‐I editing level. Supplementary Table 1. Sequences of shRNAs, and siRNAs used for experiments in this study. Supplementary Table 2. Primers sequences of gene in this study. Supplementary Table 3. Correlation between ADAR1 expression and iCCA in 128 iCCA patients. Supplementary Table 4. List of downregulated genes after knockdown ADAR1, genes with altered A‐to‐I editing level and overlapping genes. [file CPR-57-e13659-s001.docx]

**Supplementary Materials:**

**Supplementary Fig. 1 RNA expression of overlapping genes between downregulated genes after knockdown *ADAR1* and genes with altered A-to-I editing level.**

**Supplementary Table 1. Sequences of shRNAs, and siRNAs used for experiments in this study**

| Names | Sequences |
| --- | --- |
| ADAR1-shRNA #1: | GCCCACTGTTATCTTCACTTT |
| ADAR1-shRNA #2: | GCAGGGTATGTTGACTTTGAA |
| BRCA2-siRNA-1-sense | AACAACAATTACGAACCAAAC |
| BRCA2-siRNA-2-sense | GAAGAATGCAGGTTTAATA |
| siADAR1 | AGAGGUAGGUCGUAGCAUUTT |

**Supplementary Table 2. Primers sequences of gene in this study**

| Names | Sequences |
| --- | --- |
| *ADAR1* forward | 5′- AAGTCCTGCAGCGACCGTGC -3′ |
| *ADAR1* reverse | 5′- TCTCCCCGAGCCGAATGCCA -3′ |
| *BRCA2* forward | 5’- CACCCACCCTTAGTTCTACTGT -3’ |
| *BRCA2* reverse | 5’- CCAATGTGGTCTTTGCAGCTAT-3′ |
| *GAPDH* forward | 5′- CTTCATTGACCTCAACTACATG -3′ |
| *GAPDH* reverse | 5′- CTCGCTCCTGGAAGATGGTGA -3′ |
| *ACOX1* forward | 5′-ACTCGCAGCCAGCGTTATG-3′ |
| *ACOX1* reverse | 5′-AGGGTCAGCGATGCCAAAC-3′ |
| *EIF2AK2* forward | 5′-TGGAAAGCGAACAAGGAGTAAG-3′ |
| *EIF2AK* reverse | 5′-CCATCCCGTAGGTCTGTGAA-3′ |
| *IFNAR2* forward | 5′-TCATGGTGTATATCAGCCTCGT-3′ |
| *IFNAR2* reverse | 5′-AGTTGGTACAATGGAGTGGTTTT-3′ |
| *MAPK13* forward | 5′-TGAGCCGACCCTTTCAGTC-3′ |
| *MAPK13* reverse | 5′-AGCCCAATGACGTTCTCATGC-3′ |
| *ZNF655* forward | 5′-GAAGCAGCAGGGTCACCAAG-3′ |
| *ZNF655* reverse | 5′-TGTCGGTGTCCTGCACAAAC-3′ |
| *SMAD3* forward | 5′-TGGACGCAGGTTCTCCAAAC-3′ |
| *SMAD3* reverse | 5′-CCGGCTCGCAGTAGGTAAC-3′ |

**Supplementary Table 3. Correlation between ADAR1 expression and iCCA in 128 iCCA patients**

| Characteristics | | Number of patients | | | P-value | |
| --- | --- | --- | --- | --- | --- | --- |
|  | | High ADAR1 expression | Low ADAR1 expression | |  | |
| Gender | |  |  | | 0.4793 | |
| Female | 31 | | 35 | |  | |
| Male | 33 | | 29 | |  | |
| Age | |  |  | | 0.2879 | |
| ＞60 | 33 | | 27 | |  | |
| ≤60 | 31 | | 37 | |  | |
| Tumor size | |  |  | | 0.0008 | |
| ＞5cm | 42 | | 23 | |  | |
| ≤5cm | 22 | | 41 | |  | |
| Tumor number | |  |  | | 0.0076 | |
| Single | 37 | | 51 | |  | |
| Multiple | 27 | | 13 | |  | |
| Lymph node invasion | |  |  | | 0.0609 | |
| Present | 15 | | 7 | |  | |
| Not present | 49 | | 57 | |  | |
| AJCC 8th TNM stage | |  |  | |  | |
| I+II | 24 | | 38 | | 0.0133 | |
| III+IV | 40 | | 26 | |  | |

**Supplementary Table 4. List of downregulated genes after knockdown *ADAR1*, genes with altered A-to-I editing level and overlapping genes.**

| Downregulated genes after ADAR1 knockdown | CYBA，AP1M2，MACF1，NDUFA11，RPL19，EIF4G1，PTPRU，ATXN2L，SSRP1，PPP1R14B，TRIR，RPS14，BTBD2，IER5L，HDLBP，EIF4EBP1，NDUFS5，OAZ1，YBX1，CDC37，G3BP1，DDIT4，ADAR，NIBAN2，HMGA1，PCBP2，SRC，DDX1，CD44，DCTD，EIF3G，ARL2，RPL11，EDF1，TMEM238，PTPRF，MEX3D，SERBP1，NHP2，IGFBP6，FBXW2，UBA1，DYNC1I2，PRAG1，TRIM16，TGM2，RPL8，CLPP，CHID1，MCCC2，RPL22，SHROOM3，TNFAIP3，RPS2，PAK4，INHBA，THRAP3，TPCN1，TYK2，MRPL24，ZBTB11，AAMP，BSDC1，ERFE，EME2，DHRS4L2，TRPV4，ESYT1，WDR45，LIN9，PITPNM1，NUDT8，TEX261，ITGA3，DBNDD2，DAG1，RPL29，STARD8，DRAP1，PSTK，ZNF628，TMEM165，ADAP2，CHCHD6，SLC25A40，RHOT1，HMCES，C2CD4C，RPL41，RARA，UCHL3，FOXA2，CLK1，PTCH1，TRIP12，LUC7L3，PPP2R5E，PHPT1，CD2AP，CLTCL1，PLEKHM1P1，PLEKHD1，NOP10，TMC4，FAM222B，MECP2，RNPEP，HSPA12A，STAU2，DDI2，ST3GAL3，GOLGA8R，ATF3，EIF3B，ECM2，COX15，PYCR2，CBWD1，NSUN3，CLN5，TMEM44，STX17，PXMP2，RPS15A，MAP1LC3A，NPLOC4，FAR2，TMEM30BP1，ABCC3，USP32P1，SIRT4，SMPD2，ANKRD12，ATP5MF，HEATR6，RPS20P22，CCDC25，FAM174C，ODAM，TAC4，ALKBH2，TEP1，NPHS1，LAS1L，SSU72，USP51，NUP54，HSPA5，POMP，MICOS10，FAXC，TNIP3，MRPS25，DPY19L2P1，C12orf75，SPG11，CLDN20，ARAP1，ZKSCAN8P1，HM13，TNFRSF25，IGSF6，OMA1，DTX3，HSF4，U2AF1L4，GRIPAP1，ANKRD13C，FAT2，NPHP1，METTL6，FUCA2，ZNF410，NUP133，BECN1，CLPX，SFTPD，DNAJB14，SGMS1，EPRS1，ROBO3，RBM5，PORCN，M6PR，PLAU，DHRS3，JOSD1，ATP2A1，ADCY2，HMGCL，OR7E156P，CERS5，VRK1，ITGAL，LSP1P4，MDH2，PLD1，CDK14，SGSM2，KLC2，YWHAB，PHACTR1，CYS1，KIRREL3，FAM219B，ADRB1，MDM1，UBA5，DENND4B，DCTN3，PYHIN1，ERCC5，ACSM2B，TRMT61B，NRXN3，PPARA，MTCO1P11，ABCC6P2，ZNF391，RPP30，CSF1，TACC2，ATP5ME，ZBTB8OS，ARGLU1，GFUS，SELENOW，PHF14，DAPK2，ZNF787，S1PR3，MVB12B，CCNG2，SV2B，FAM86DP，CPHL1P，LIME1，PSMA4，SRSF10，CCL24，NCBP1，CCNG1，PSMC6，RBIS，SCNN1D，LARS1，TGFB2，TSFM，MTND4P14，MPZL1，TDRD15，KANSL1，UBTF，IMMT，SRSF5，SDAD1P1，SAMD10，PCLAF，CALM1，RPL30，IGFBP3，CLASP2，LSM4，STK31，SLC22A20P，LCAT，CEP295NL，SLC25A14，RTKN，FAM237B，POLN，IMPG1，SREBF1，ACTA2，ADAM21，STYXL1，KHDC1L，IQSEC2，FOXO6，PFDN6，NBAS，HSPD1，GDF9，SLC22A18，USF2，GUSBP1，CYP51A1，SLC25A43，LTA4H，RYR3，ETV1，PUM1，ESYT3，UVRAG，TMEM222，FKBP9，CSNK2B，MKNK1，ADD3，VNN3P，UBE2N，TXN，STARD10，LMNTD2，PALS2，RSF1，CDC6，CBY1，ACTR8，SLC35B2，MICU2，ARHGEF40，KALRN，ATP6V1D，PTRHD1，AMZ2P1，HMGN5，SCARB2，C1orf109，DROSHA，MITD1，COMT，SLC25A27，DHODH，TBC1D3I，KIAA0100，TMEM218，PCDHGA9，RNF8，FAM136A，SBNO2，IGFLR1，EIF3H，CXXC5，ERICH2，PPP1R12B，YARS1，TRAPPC12，PLXNB3，ARHGAP24，KMT5A，P3H3，TIMM21，NAPA，ALDH3A2，PTK2，SLC39A6，FAM72A，RSRC2，TFAP4，ZFP41，RARG，CHURC1-FNTB，RREB1，LRP2BP，CLEC17A，BNC2，CDKN2A，ZDHHC17，PRODH，PRR5-ARHGAP8，PYM1，ZNF721，NIFK，TDRD6，C11orf24，LARP4B，CCDC9B，GLS2，COG3，XNDC1N，PI4KB，YJU2B，GAS8，PSMB4，ANK2，COPS5，BCL2L12，PPA2，SCAND2P，SLC16A4，DEDD2，MED19，SMAGP，ZNF497，PKP4，SRPRB，DNAJC22，SLC5A6，AKT2，ZNF692，GDPD1，BBS1，SH3BP4，ZNF717，CORT，SLC38A5，CHERP，FHIP1B，CARHSP1，PIH1D2，DPAGT1，FABP3，PPIP5K1，FIGN，DNA2，TFEB，ACTB，GSE1，NAXE，RFK，CLIC3，SPAG7，PROSER3，PRDX5，CEP95，RRM2，XPO7，SLC23A3，TBK1，PKD1，MT1F，MFSD11，IP6K1，CYRIB，ADGRV1，DPY19L2P2，POLA1，MAN1B1，CC2D2A，SMCO4，SLCO1B7，ETFDH，TNFRSF12A，MTHFD1L，EMP3，HMGB1P3，RSRP1，SLC39A9，PTCD2，PIP5KL1，ADAMTSL5，NDUFS7，BBX，SHOX2，TUBBP5，KIF19，SMPDL3B，TAGLN2，PRKAG2，PCDH9，FOS，SPNS1，LIMA1，RPL38，PANO1，MIGA1，LBHD1，WDR25，BRAT1，CHCHD5，EOLA1，PCDHGA8，TFIP11，RPL10，KRBOX5，SLC25A35，SEMA6C，PLEKHG4，BIRC2，TAOK3，CHP1，AKT3，GNA12，ZMYM3，ADCK1，MAGIX，FCF1P7，ACD，TMED6，RAB3A，PPP4R1L，USP15，AKAP7，ZC3H18，SLC39A14，LRRK1，SEC14L1，CTNNBIP1，DGCR6，SGSM3，CHD6，RNF167，ESPL1，LRCH3，TRIM8，TCF7，IMPACT，CRMP1，ALG5，MLPH，RNF40，SIRPA，CPNE7，EBNA1BP2，CDCA3，ASPSCR1，RPS6KA5，DNAH5，SAP25，SDHAP3，CFAP52，FANCL，GFY，SLX9，TMEM45A，KXD1，LRPPRC，SARDH，NDUFAF6，GNB5，IFTAP，GTPBP4，MRPS18A，ZNF138，METTL1，GTF2IRD2B，TUT7，INPPL1，IFT88，LAT2，AURKB，MYH9，MMS22L，RCBTB2，RPL23AP53，ZNF565，PTBP3，ZFP69B，SIRT7，REXO1，RAP1GDS1，CRYBG2，PPP1R26，DDX39A，RPE，OSR2，ITFG1，MTHFSD，PSMA3，NRBP2，MLF2，MRPS27，USP10，CHCHD7，PIK3C2G，ST6GAL1，TSSK6，NADK，GOLGA8N，ARID5B，THOC1，RTBDN，TAPBP，TEPSIN，ARRB2，MFF，TUBA1A，PTGFR，KIF20A，NVL，CROCC，TBC1D31，ZFAND2A，TCF25，CTAGE4，TENT4A，WFDC2，PTP4A2，SEMA4G，CTSC，TSPO，PLEKHM2，NFS1，TIMM9，SBDSP1，NDUFV2，VPS8，CDK2，FBXL3，PILRB，CYHR1，KLHDC9，INCA1，ARRDC1，ADAMTSL1，ANXA8L1，LETMD1，SERF2，ZNF558，AKAP1，PNPLA3，ARPIN，VPS37A，MRPS11，NT5C3B，ESPNL，SS18L2，ELL3，HIVEP3，SRRD，APBA3，CHD2，WT1，NSD2，PKP3，CPT1C，EIF4B，APCDD1L，GLS，PDPK1，DIMT1，GCFC2，SON，SNRNP25，UROS，LAYN，DUX4L50，ZNF619，STIL，FBXO9，ZNF383，CRELD1，LRIG1，KIF21A，LRRC24，IMMP2L，CPNE1，ILF3，SEC31B，TSSC4，RAB35，RNF214，PLK3，PLOD3，CD320，BCL6，CERT1，METTL26，DPH1，DNM1L，EXOSC1，CERS2，CPSF7，CCDC24，DYM，DYNC2I1，MALT1，MXD3，SLFN12，TLN2，TK2，PXN，SLAIN2，PFKP，THAP6，MFSD14C，TRIM66，CYB561，ACAA2，NT5C，FAH，SEC31A，SPRED3，SLC7A4，SLC52A2，IGFL3，NTAQ1，TRMO，LIG3，LSM14A，CAMK2G，HOMER3，ASB18，MPPE1，CYP1B1，TRPS1，IQCH，ZFTA，RBM8A，TMC8，SEC22A，LAMTOR5，TROAP，SRRT，ZNF792，NEMP2，HSP90B1，TMEM134，GREP1，PHF20L1，NIPSNAP2，EIF3J，TMEM63A，TMEM98，SVIP，GLDC，TAF4B，FIG4，PDLIM2，TRMT10A，ARL6IP4，GALNT11，AGBL3，DYNLT2B，MMADHC，FUT8，ANO1，C3orf18，GALT，SMARCD2，RPL36A，CYBC1，C17orf49，KATNBL1，TEDC2，DPY19L2P3，FIS1，CCDC66，MRPL3，C7orf25，ACBD4，GPCPD1，CCDC122，DHX38，TMA16，CIAO3，ATP6AP2，ZNF701，SLU7，OTUB1，PARP1，MAP3K7CL，ATF1，ERCC4，PIP4P1，RABEP2，PROM2，LPCAT1，TBL1XR1，TBRG4，BTF3，FIBCD1，AAR2，TRMU，MAT2B，SNPH，AFDN，MMS19，SMAD3，ACSF2，TUBE1，TMEM147，ZBTB24，FAM149B1，PNPO，NPIPB5，ACAD9，CLP1，ECSIT，RANBP17，ST6GALNAC1，TMEM170A，SUCLG2，TP53I13，TPR，ALG3，CLUHP3，LYRM7，USPL1，FANCA，ZNF252P，ERI3，PMS2，FAAP20，LONRF3，SLC1A2，TPRA1，TBC1D25，PIMREG，ANKRD13B，NUP62，PDP1，GPR146，ALAD，TPI1，ZC3H11A，BUD23，MBD1，PARP16，MAMDC4，RBMS3，SMPD3，NOMO3，PPCS，LRRC49，PTPN22，WDR90，MMP19，TMEM260，CD99P1，METTL27，MICOS13，C4orf36，CHMP1A，WNK1，OLA1，MAPK13，DAP3，LRRN2，MZT2B，SORCS2，PTPN2，TATDN1，CNKSR1，PRPF40B，MCTP2，DDB2，OXLD1，KITLG，ITPR1，RANBP3，TRPT1，CLASRP，AP3B1，NHLRC3，TXN2，ZNF833P，MTCH1，ATRX，DUSP3，TMEM185A，FAAP24，GNB1，PLEKHA1，SHC1，DHRS7，SLC44A3-AS1，EPM2A，SLBP，HPSE，EIF4E3，ZNF600，FBLN5，KCNIP2，CCDC59，HELLS，ERMAP，PLGLB2，PDHA1，GTPBP3，TNPO2，KLRK1，MTFP1，FBLN1，GMPPA，RB1CC1，KCP，ZNF696，MVB12A，GSTCD，PRSS22，MYCBP2，KCNG1，WRAP53，MRPL45P2，STK4，CAD，OGFOD3，KNDC1，SENP6，CDC23，HNRNPC，HES6，SETD5，GNPAT，ANXA2R，ADCK2，ST7，MLH1，OSBPL5，CA12，FDFT1，NETO2，PRKAR2A，FOXRED1，PPM1F，MED20，HS3ST1，LRCH4，MKRN2，PACS2，RGL3，EME1，FUZ，GALE，FBXO15，CKMT1B，OXSM，ZFY，MBD5，AMMECR1，SMAD7，POU5F1，MAD2L2，LUC7L，STK16，PRELID1，GNA11，MYSM1，BMPR1B，CFAP119，HGSNAT，AAMDC，GK5，YIPF3，HYAL1，DNHD1，ERMP1，ESYT2，CDC14A，FBXL13，LRRC23，TMSB15B，ARHGEF1，ZNF747，ADSS1，CLEC2D，TCERG1，VPS4B，SSH3，CLTA，ABHD11，BBS4，CAVIN3，ZNF280C，KIZ，TNPO3，TIMM23B-AGAP6，SPINK5，NPRL2，GOLT1B，PABPC1L，DZANK1，B4GALT3，UBP1，CHD7，NME1-NME2，DENND1A，OSBP2，ING1，ID2，DOCK6，NFKB2，HDDC3，KLHL7，SMPD4，C11orf91，FRG1HP，PTBP1，PTPN4，SYS1-DBNDD2，SEC61A1，UBE2C，ULK1，BAX，CTSH，AGFG1，IL32，IFNGR2，OPRL1，FHOD1，RPS6KB2，CAB39，SACM1L，SOS1，PRSS8，TMEM43，LAMTOR2，FAM204A，RABGAP1L，RGS7，PPP1R37，ZNF524，SDF2，EPB41L1，GNE，RNASET2，SLC66A1，STXBP2，TMEM138，C1orf50，NSMCE4A，GCA，ZFYVE27，ATP5F1A，TASOR2，SLC6A9，ANKRD23，CIT，S100A8，ZNF821，GRB7，SKIL，MSRB2，IGSF8，RRP1B，AFTPH，CCT6P1，POU2F2，GPX1，MED26，STAG3L4，ZBTB41，LRP8，METTL3，GEMIN8，CAPN5，HERC2，ZNF133，SPRY2，CGB7，EBAG9，KNL1，NDUFS2，PARN，NCOA6，TBX6，HLTF，NCAPD2，CTC1，GAK，RCC1L，TCEA2，ANXA3，RAD51，RAB5C，SGK1，TLE2，ATG4B，FUT2，CRYL1，EIF4A1，EML2，EIF4E2，GALNT1，PDCD2L，GABRE，NPHP3，PARP3，NDUFS8，POLR3H，SUB1，HROB，AGPAT3，NBEAL2，FADS3，SPG21，NOTCH2NLR，ZNF384，SPATS2L，HOMER2，SCNN1A，NDUFV3，PPOX，GPI，E2F6，LRRC51，NOL8，XKR6，ZFYVE28，CALM3，COL21A1，PDXK，AAAS，SLAMF9，ANKRD46，EPS8，NGEF，HACL1，MARK4，GGA3，UNC119，KIAA0586，SEC61B，CLEC16A，LMNA，CNPY2，OSBPL10，FBXO46，ZDHHC1，MRPL48，UBE2Q1，KIF11，CSNK1A1，VPS37B，MAD1L1，ARSA，SNX7，ENKD1，GRB10，IL17D，MIIP，CNTRL，LETM2，TMEM229B，NEK11，USP21，VEZT，PQBP1，SH3TC2，TARBP2，RAD52，KMT2D，DCXR，AKTIP，KIAA1755，SLC35A1，PAIP2，METTL7A，SPEG，MFSD10，AGAP6，LMTK3，DLG4，CDC45，RAB1A，AHCYL1，DCAF6，FTSJ1，PLCG2，HADHA，CD164，ITPRID2，FXYD5，TMEM25，CFL1，ACY1，TMEM262，NACA，DENND4C，RITA1，GPHN，AGPS，SEPTIN7P2，KATNIP，GOLGA2P7，R3HDM2，TPD52L2，AGO1，REPS2，ABCC5，FAN1，FAM86B3P，POLK，ALDH3B1，POLD3，LINC01145，SORBS2，NDFIP2，SHKBP1，HIVEP1，IRAG2，LARP1，XYLT2，NFKBIA，SNRPD2，ITM2C，ZNF3，FBXW7，UPP1，SNX15，ACYP1，FLYWCH2，ADGRA2，IMPDH2，LMCD1，TATDN2，RAD50，CTBS，NUP50，ZNF229，HIC2，FLNA，TPCN2，CBLB，NOD2，NARS2，POLA2，WBP1，NBN，AHCY，UBR3，EPC1，SLC25A38，KPNA2，DIP2A，VASH2，PDE4C，LIN54，PAF1，DDX17，COMMD3-BMI1，ISOC2，PIGU，CSAD，ARHGEF3，LZTFL1，ZFAT，FDXR，FGFR2，LIMCH1，AP2S1，SLC66A3，RNF20，GKAP1，POMT1，GHDC，SERPINF2，SENP2，SPATA33，GIT2，RAB11B，PCGF3，ZNF611，QRICH1，TPM4，TSPAN31，KLHL22，TTC39A，FGGY，CCDC115，BARD1，JUP，LTBR，NPEPPSP1，SPIN1，PSMC3，GPRC5C，XRCC3，PHYHD1，TAFAZZIN，MOXD1，AP3S2，CCT5，DDIT3，RGS17，IQCE，HMBS，CENPX，HACD3，GPR155，WNK4，NET1，C1orf112，PRMT2，CARF，PES1，DPM2，STAT2，SNX24，NT5C2，ITGB7，SKP2，BCL7B，NPEPPS，NEK9，TDP2，ZNF525，SEPTIN7，CRPPA，B4GALNT3，RHOQ，MAN2C1，ASNS，PIGQ，ODF2，ZDHHC16，MRM3，ABCA13，CDK20，SYNE4，FKBP2，ARFRP1，GNAI2，ADD1，ALS2，INTS8，GLB1L，SSR4，KYAT1，ZNF365，DPF2，CASP9，TBCE，RAP1GAP，LIN52，EFNA1，MFSD4B，GRAMD1C，RBPMS，TRIM25，NECAB3，RNF123，SSR2，CNGA1，C1orf159，GSAP，ERO1A，KIAA1109，CIITA，POLR3D，KIFC3，SYNE1，VPS45，LIMD2，PAMR1，MSMO1，DYRK3，BDNF，LSM12，PDE8A，MTRR，AXIN1，ASAH1，DISP1，SIDT2，RAE1，C2CD2，PFDN5，ELOA，FAM216A，ZNF580，PPP2R5B，CCT8，NISCH，TMEM214，UBE2I，PPP2R3B，ZNF276，ADCY6，ZNF343，FAM161B，MEN1，ANO9，ESRRA，POLE，ZNF146，PIK3CD，PPFIBP2，RNASE1，PRICKLE3，C14orf93，SLC35E1，AP4B1，SF1，TMC5，POLM，RCC1，CDK2AP1，ATF7IP，RUVBL2，B3GALNT1，NAV3，SLC41A1，CDON，ARHGAP26，GALNT14，EXOSC5，METTL14，ST13，SPIDR，ATRIP，MUTYH，PWWP3A，ALDH4A1，MYL12A，IFNAR2，TIMM44，SNAPC2，GSK3A，RAD51B，NHEJ1，PITRM1，PMS2P4，HSD17B1，KANSL2，UBAC2，PHGDH，TPD52L1，JMJD4，FAM120A，ZNF510，MKS1，PGAP2，HEATR3，STIM2，RFNG，RARS2，NKRF，MYEF2，CDK5RAP3，ABHD10，TTC37，BRMS1L，U2SURP，CSNK1G2，EXOSC7，BRCA2，ARSB，DPH2，ACSS2，MAPK3，MRPL55，STUB1，PRKCD，SDHAP4，ATAD3B，RTL10，SHMT1，SORBS1，COASY，USP53，MYCL，DDX54，LRSAM1，SNX1，BICD1，EPS8L1，PHOSPHO2，ST6GALNAC2，MON1B，AMDHD2，LYSMD4，DEAF1，RRP1，ADAMTS9，TIPARP，TEX10，KCNQ4，EWSR1，GPR35，MCOLN3，ANKIB1，CHMP3，SLC38A7，SNAP25，TOGARAM1，NCBP2，NSMCE2，TMCO6，CDK16，POLR3C，L3MBTL1，MKI67，CDPF1，KIRREL1，CEP126，PDCD6IP，RBM41，LRBA，SLC1A3，FOXM1，MTMR9LP，FER1L4，C1QTNF1，ZFAND2B，COLGALT1，ATP6V1E1，EP400，AMMECR1L，SLC4A11，TRIM56，STAMBPL1，KDM1A，HERC2P2，CEP41，PHAX，DDX18，ATP5F1D，TRIO，SLC22A5，DNAJC4，SYT7，LYRM1，CCDC18，ERCC8，ERGIC3，C6orf136，MIA2，SNX5，ACTR6，VLDLR，SAC3D1，HMBOX1，IQCK，NF2，SLC41A3，ZBTB44，QKI，FASTK，FBXL2，MGRN1，GNL3L，UBA52，RABL2B，ATXN2，TYMS，JADE2，BBIP1，DNAH1，PGM3，SYTL1，XPNPEP1，RPGRIP1L，SLC25A32，BBS9，ACP2，PTPN6，ERGIC1，MVK，IFT22，EIF2S1，RAP1B，MTMR11，ZSCAN31，MTHFD2，KLHL24，PPIL2，EIF2B2，PHF21A，WDR73，TP53I3，ZDHHC5，DDX41，GON4L，RAB4A，ACOT13，BRPF1，MAP3K14，MAZ，TMPRSS3，TIRAP，CEP57，APBB3，TCTN3，KLK10，PSMD4，CIBAR1，DDHD1，KTN1，STK26，SIAE，MAPKAP1，ITGB5，MGAT1，RMDN3，LSM14B，CD302，MAP2K3，NONO，SYS1，RBM42，PF4，ADAP1，PIAS2，TLR5，CHKB，AMFR，SPG7，PHF6，UBE2F，MPST，HCN3，ZDHHC6，KIF1C，PPAN，XRCC4，RWDD4，MAPK14，RNF220，PTEN，POLG2，SUPT20H，P4HB，HR，TMEM17，KANK2，SLC25A22，HSPA9，ZMPSTE24，VPS72，UTY，CASTOR3，LINC00680，NSFL1C，TPK1，AUTS2，MTMR3，MPND，ZFPL1，CHFR，NDUFAF7，PASK，TGFBR1，PLEKHA5，ABCD4，PLEKHJ1，MBTPS1，ANKRD18B，TLE3，ARL13B，ERLEC1，TMEM69，OSCP1，NSD1，SOS2，ZNF652，ARSD，AMPD3，HNRNPUL1，PDE8B，ABCC4，PKP2，RASAL2，ZC3H4，FOXP1，CAPRIN2，ETF1，TRIM3，CPEB4，MIER1，TAF1B，MEF2A，RAB2A，C2orf74，LAMTOR4，ELF1，VILL，AKR1C3，HMGN1，CNDP2，INF2，AIDA，STARD3NL，ZBTB45P2，PPFIA1，DAPK1，EIF5，DCAF7，SF3A1，GUK1，IQCJ-SCHIP1，LCK，RAB11A，CYP27B1，TDRD3，DONSON，MAMLD1，STEAP2，NNMT，GAS2L3，ZP3，PPIG，CD276，APOL2，PCYOX1L，THTPA，SSBP1，ZNF211，DVL2，ANKFY1，TUBGCP3，PPP6R2，ACTR10，ZNF395，MAPK12，ZNF266，CCDC120，KAT5，HMCN2，C7orf31，CNOT1，RPL7L1，CAP1，DPH5，CEP135，ZCCHC17，ZNF160，SEC23B，TECR，DAPP1，ETFA，CUEDC1，ACADVL，SAT2，CDK11A，IKBKG，SUPT5H，UMAD1，DAB2IP，ZNF200，ZFYVE16，RXRA，NUDT16，SMNDC1，ASH2L，MDN1，HDAC10，TXNDC15，CFH，APTX，MORC2，MAFF，ZNF704，ZSCAN2，GGA1，USP45，EGLN2，KLHL5，RAB3GAP2，TANGO2，SRPK2，EMD，SLC4A2，TFDP2，HTR7，SH3D21，ADCY7，ALMS1，THYN1，MYRF，UNC5A，STX1A，HAPLN3，EDA，CASP3，TUG1，UBE2D3，STRN4，ASB1，TADA3，OAF，KDM5D，MGST3，SLTM，SEMA3B，SNU13，ITFG2，DKC1，COPZ1，WWP2，TUT4，SLC29A2，DIAPH3，ALG1，BRF1，MOB2，SNX11，ITSN1，MRPS2，PXK，FBXO32，CALCOCO1，KLF4，WAC，RIT1，SEPSECS，NIPAL2，SCARF2，ADGRB2，TTLL1，MARK3，CES3，TBC1D2，APLP2，FGD3，AVL9，ZNF45，TBCB，PDK2，RPP38，CEP120，CEP89，AFAP1L2，ACOT7，PISD，ZNF527，HINFP，NAXD，ZMYND8，EPS8L2，CPNE2，ELMO3，MSANTD4，ZSWIM8，RNF41，IDE，SLC25A1，MTCL1，SERPINA1，DGKZ，RFX2，RSRC1，SF3B1，TTLL3，PTGR1，SNF8，LONP2，EIF3E，FTO，TBC1D7，XPNPEP3，PIEZO1，AIFM1，TM2D3，GRAMD1A，PDIA3，PPP2R5C，MCM3，TLR4，BCAS3，CHAMP1，CEP63，PHETA1，FGFR1，AMPD2，MAP4K2，GPR68，RAI14，PUS7L，DHRS13，ARFIP1，C1QTNF6，KDM5B，PSEN2，GBF1，MECR，MARK2，ZNF419，FRMD5，COMMD9，NAA15，FIBP，FANCB，RAPGEF1，PEF1，CCDC88A，SCFD1，NGLY1，RHOC，PEX10，SELENOT，SLC12A9，MCPH1，TAF8，UCK2，VCPKMT，CCNL1，ITPA，CDC42BPA，TMEM67，INPP5F，PSEN1，KLC4，KCTD17，CHD4，DDX42，SH3BP5，C11orf54，RHOA，SKA2，SLC26A6，HIPK3，ABL2，SORBS3，RPS3A，ACSL4，IKBKB，ITGB4，STRN3，ABCB8，RAB34，SLC25A46，ZNF473，TSPAN17，CCDC47，DMKN，SSBP3，NCDN，MAP2K1，DMPK，FGF2，SRSF6，KIAA0319L，ANAPC15，AARS1，FBXL12，NR1H3，ZW10，NCOA1，TPM3，TRNT1，WDR20，ACE，PARP6，FAM156A，ZNF292，NFXL1，SLC38A6，SSNA1，ERC1，RAD21，HIVEP2，RPL26L1，CDC20，MTHFD1，CRIP1，FOXRED2，TSEN54，BICDL1，PEAK1，NOMO2，ARID2，HARS1，RCHY1，CDK4，WDR31，EEF1D，SENP7，FBRS，TGFB1，NLE1，RALGDS，ARMC10，BRCC3，IL17RC，ACVR1，SLAMF7，IMP4，C9orf78，COPB2，EPB41L2，RPL13A，FANCM，TMEM120A，ITGB3BP，ZNF608，PPP1R15B，DST，LTBP3，CEP192，MOGS，TYW3，SLC19A2，RBM6，ILVBL，BID，SYNE2，DYNC1H1，ARMC9，GPAA1，NFYC，CLASP1，PGM2，SMARCA4，TMEM52B，PCCB，ZKSCAN5，SH3YL1，C19orf25，FHL2，TRIM37，MYO1E，FHIP2B，ACOX1，GMFB，DCAF4，RIC8B，NAA60，LARP4，MACO1，PABPC4，SEPTIN6，TRAFD1，DSN1，ELP4，TBC1D22A，MARCHF2，SH2B1，ACTN4，SLC30A6，CYB561A3，ARHGAP45，FDPS，CIZ1，RTEL1，ATG9A，POMGNT1，JAK1，RIPOR1，CGGBP1，SCYL1，ZBTB7B，ASPM，ZMYM5，SLCO1B3-SLCO1B7，SEC63，CTDSPL2，NQO1，RPS6KC1，TESK1，SNX21，USP39，NFKB1，VPS53，VPS52，SHANK3，PGRMC1，UBXN11，ATF6B，DCPS，ING4，CSPP1，STAG1，IL11，MCM8，SUMF2，SLC3A2，SLC12A8，DHCR24，STX16，KLC3，ZNF148，SEC14L2，ENDOV，BANP，ZNF655，CCDC124，AASS，EXOSC8，POP1，SETD2，ETS2，PREB，ICA1，BCAP31，LGALS1，NRDC，SEC16A，GNAI1，HPS1，CMTM3，MIB2，RFXANK，NAPEPLD，METTL17，LYPD6B，WDR62，ERCC6L2，EIF6，WFS1，TBCK，CASP8，DLG3，QARS1，ST3GAL6，TPP2，SMARCD1，NCOR2，GMEB1，TERF1，INTS4，ADSL，BLOC1S2，ZNF184，SH3PXD2A，SMAP1，OTUD5，COX5A，NUTF2，PARD3，CLIP4，FAM8A1，DMTF1，KPNB1，PPP6R3，RABL6，DUS1L，RBBP8，CYBRD1，AKR1A1，IFT122，ARHGDIA，PID1，CLIP2，RNF19A，NAGK，SIPA1，TOM1，TRMT2B，RAP1GAP2，LARS2，EPB41，MATR3，CUL7，NUMA1，TMPRSS2，TAB2，PTPRR，RASGRP1，TSHZ1，CLPTM1L，IGF2BP2，EXOSC2，IL20RA，CYB561D1，ZNF438，IRF3，UPF2，NFAT5，KIF22，LZIC，FKBP1A，SUCO，BTBD10，TOX4，POLD1，DNM2，CFTR，SUN1，CACNB3，INTS9，NDE1，CDC73，R3HCC1L，ELOVL1，EIF1AD，MED24，CTNND1，CYREN，ANKRD11，ADAM17，SHROOM1，DUT，ARID1B，STAG2，SUGP1，TRIM2，CUL4B，LASP1，BRIP1，TAF1C，EXT2，FAM219A，KRIT1，UBAP2，SLC19A1，NDUFB5，PLEKHA7，VPS26B，AKAP8，VPS13B，NCAPD3，DCAKD，PGRMC2，BCL2L1，DHX35，DDX5，CABIN1，ZNF362，SPINT1，SEPTIN9，AIFM2，SPECC1，TCEAL1，GLB1，ZNF142，NIPBL，FBXO7，ODC1，ITGB1，MED16，SLC44A1，LRRC75A，ASL，ZNF518A，NOLC1，KLC1，NUP210，XRRA1，TOM1L1，C6orf89，AHCYL2，WNT5A，RIC8A，PLAGL1，USP19，SYNJ1，ZDHHC4，SNX2，NAMPT，ELAC2，EIF2AK2，DDB1，FMR1，TCF7L2，ANGEL2，WDR18，MBNL3，MAP4K4，KDM5C，SBF1，PLEC，RIC1，PRR13，ARID3B，ELMO2，PTGR2，STK40，TMEM161A，POLR2H，SLC11A2，ZNF841，CTNNA1，NFE2L2，INPP4B，TNFAIP2，RIN2，CISD3，GPS1，DCAF17，TJAP1，TOMM40，PARK7，MARS1，ABCF2-H2BK1，IRF1，DSE，CDK5RAP2，SLC35F6，HMGB1P5，TSKU，HECTD1，NDRG3，EML3，LZTS3，GLIS3，GCSH，GAB1，BBS2，USP8，PRRG1，N4BP2L2，PIK3R1，PHLDB1，MSLN，IGSF9，TTLL4，POLR1D，ARMCX2，GNL3，SPATA13，ABLIM1，LCORL，CCND1，BABAM2，SLC43A3，RUSF1，KIAA0895，ZBTB18，HNRNPDL，MYO6，CEPT1，GOLGA2，DOCK9，NECAP1，PFKFB3，KMT2C，OPTN，C8orf33，PRKAG1，SETDB1，SLC38A10，ZNF638，KIFAP3，MST1R，ZFAND6，IFT140，PTPDC1，MYD88，PNKP，ZNF700，COPS7A，NEK4，HNRNPA1，SLC35A3，CEP104，HNRNPA2B1，WIZ，CDC42EP3，NSA2，SCYL2，PNPLA6，RNF170，ITCH，EIF4G3，GATAD2A，FOXJ3，LIG4，SH3BP2，MSH6，PHB1，CCSER2，TEX2，DENND3，SVIL-AS1，SPSB3，AHCTF1，WASHC5，EP300，AAGAB，GNAS，PIGO，UBB，TRIT1，OPA1，MAPKBP1，PARG，ALDH7A1，HNRNPH1，KIF23，AKT1S1，AKAP9，MCRS1，TSPAN9，SRSF1，RBM12B，HSP90AA1，MED13L，RBM10，RPRD2，NUP88，AMZ2，VMP1，ZNF286A，BAZ2A，KDM2A，AKT1，RNF38，PCSK7，ADK，SYNCRIP，CAMKK2，AGAP3，BMP4，RALY，TXNRD2，ITGB2，SIPA1L1，PHLDA1，XPO1，NHS，UHRF1，DEDD，ATE1，THOC2，KMT2B，ARNTL2，G6PD，TRAPPC1，TRAF3，PRKAR1A，MAPK9，C19orf48，FARP1，PNPLA8，ATP2C1，ARHGAP21，NFE2L1，TRIM16L，ESF1，SZRD1，TRABD，HDAC6，NUP58，DDR1，DYSF，MAP9，SOCS7，CRAT，NEMP1，PTPN13，RAF1，TXLNA，SSH2，CTNNB1，MLST8，CALR，KPNA4，SMARCAL1，DNAJC7，TGFB1I1，NUMB，OS9，YAP1，RAB43，SLC12A4，SEPTIN8，PIGT，ZKSCAN1，LAMB2，ECE1，SPATS2，MAP4K3，SLC16A7，FAM102A，WWC1，TAB3，PLAT，MEGF8，MGAT4A，KCTD2，CSNK1D，RPL18，RRBP1，RALBP1，MYO9B，ATAD3A，ARHGEF28，CSDE1，CAST，RBM47，WARS1，TRRAP，MED29，STMN1，COPA，ASH1L，EHMT1，REPIN1，YWHAZ，DHCR7，IPO5，S100A16，DICER1，MAGED2，HNRNPU，ACTN1，PLXNB2 |
| --- | --- |
| Genes with altered A-to-I editing level | H2AZ2，NUP155，C15orf48，COL4A5，SLC33A1，INIP，FADS1，BRCA1，HOOK3，MAP3K9-DT，METTL2B，TRUB2，AP4B1-AS1，EMP2，EMC1，ALCAM，SNX19，LIMD1，TLCD2，ANKRD10-IT1，CBFA2T2，GP6-AS1，HP1BP3，ZNF432，FAM126B，BCL2L15，CHMP6，SPCS3，ALDH6A1，FGD5-AS1，CTSB，CENATAC，XIAP，PEX26，LINC02977，PSMD12，GPR157，CEBPZ，KAT8，PTPN14，ACOT9，THEM4，XPOT，ARPC1A，DTWD2，RSPH3，MAVS，MAPK1IP1L，GTPBP2，CHTOP，FAM111A，FAM20B，GSEC，BRI3BP，PLBD2，AP1S3，COX16，LINC02987，PFAS，FBXO34，DMAC2L，SLC35F5，AXL，BPNT1，IL10RB-DT，SPPL2A，BIVM，ZNF808，BBOF1，CHUK-DT，LGALS3，PABIR3，RBSN，PDP2，FCF1，APOOL，ZDHHC20，RPL28，DENND6A，SRD5A3，MYDGF，MED21，SLC26A2，RNF24，SYNJ2BP，POLH，PLEKHA2，LIMD1-AS1，ENO1，UBE2A，PANK2，MAP4K3-DT，RPL37A，STAG3L2，OIP5-AS1，SWI5，ZNF814，TMEM192，SFT2D2，STEEP1，CLTB，SLC35E2A，MRPL30，HINT1，CEBPZOS，TMOD3，SPC24，XRCC2，NDUFB1，ALOX12-AS1，UGGT1，RDH13，SNHG4，LYRM2，APOL6，ANKRD10，EMC1-AS1，CASP10，SYNC，SLC12A6，MORN1，GPATCH2L，TIRAP-AS1，CENPN-AS1，VPS41，GPALPP1，FADS2，SLC25A25-AS1，MAGT1，ARNTL2-AS1，TMPO，NBPF20，UGDH-AS1，RRP36，CTSS，PMS2CL，RAD51-AS1，ZNF587B，CWF19L1，RIF1，PSMB2，CSRP1-AS1，RBBP4，WDR4，TMED5，CCNYL1，TNFAIP8L1，PLCD4，PPIA，CD46，CENPN，RCAN3，STYX，CINP，TTC7A，TADA2A，ZNF621，DCAF16，MRPS30，STAG3L3，PRR11，RAB17，ANXA5，POLR2D，GATD1-DT，NMRAL1，PLCXD1，PHACTR4，PGAM5，TAF6，PML，ZYG11B，C19orf71，GINS4，ZNF69，H6PD，GNPNAT1，SIL1，ALOX12P2，STAG3L1，SANBR，CCL28，SRD5A3-AS1，RPL27A，SMIM14，MFSD12，HFE，ZNF587，SRFBP1，GNRHR2，NBR2，GPAT4-AS1，DDX58，SNRPD3，VOPP1，PSMA8，F11R，RPL30-AS1，NUP43，DNAJC24，EPB41L5，ATG14，NBPF15，CLTA，ZNF655，TMEM170A，TRIM56，GK5，TRMT2B，EIF2AK2，BRCA2，TEP1，ZNF142，GNE，XPNPEP3，MAPK13，CLPX，NPLOC4，ARSD，DCPS，TIRAP，IFNAR2，MALT1，MATR3，TXNDC15，DNAJC22，PRKAR2A，ACOX1，TMEM165，ARNTL2，CCDC120，GAS2L3，RPL30，PUS7L，PIEZO1，GNL3L，NDUFV3，ZNF701，KNL1，ZNF841，HELLS，XKR6，RBMS3，SLC35A3，RBM8A |
| Overlapping genes | CLTA，ZNF655，TMEM170A，TRIM56，GK5，TRMT2B，EIF2AK2，BRCA2，TEP1，ZNF142，GNE，XPNPEP3，MAPK13，CLPX，NPLOC4，ARSD，DCPS，TIRAP，IFNAR2，MALT1，MATR3，TXNDC15，DNAJC22，PRKAR2A，ACOX1，TMEM165，ARNTL2，CCDC120，GAS2L3，RPL30，PUS7L，PIEZO1，GNL3L，NDUFV3，ZNF701，KNL1，ZNF841，HELLS，XKR6，RBMS3，SLC35A3，RBM8A |
